# Understanding inherent influencing factors to digital health adoption in general practices through a mixed-methods analysis

**DOI:** 10.1038/s41746-024-01049-0

**Published:** 2024-02-27

**Authors:** Lisa Weik, Leonard Fehring, Achim Mortsiefer, Sven Meister

**Affiliations:** 1 Health Care Informatics, Faculty of Health, School of Medicine, Witten/Herdecke University, Witten, Germany; 2 Helios University Hospital Wuppertal, Department of Gastroenterology, Witten/Herdecke University, Wuppertal, Germany; 3 Faculty of Health, School of Medicine, Witten/Herdecke University, Witten, Germany; 4 General Practice II and Patient-Centredness in Primary Care, Institute of General Practice and Primary Care, Faculty of Health, School of Medicine, Witten/Herdecke University, Witten, Germany; 5 Department Healthcare, Fraunhofer Institute for Software and Systems Engineering ISST, Dortmund, Germany

**Keywords:** Public health, Health services

## Abstract

Extensive research has shown the potential value of digital health solutions and highlighted the importance of clinicians’ adoption. As general practitioners (GPs) are patients’ first point of contact, understanding influencing factors to their digital health adoption is especially important to derive personalized practical recommendations. Using a mixed-methods approach, this study broadly identifies adoption barriers and potential improvement strategies in general practices, including the impact of GPs’ inherent characteristics – especially their personality – on digital health adoption. Results of our online survey with 216 GPs reveal moderate overall barriers on a 5-point Likert-type scale, with required workflow adjustments (M = 4.13, SD = 0.93), inadequate reimbursement (M = 4.02, SD = 1.02), and high training effort (M = 3.87, SD = 1.01) as substantial barriers. Improvement strategies are considered important overall, with respondents especially wishing for improved interoperability (M = 4.38, SD = 0.81), continued technical support (M = 4.33, SD = 0.91), and improved usability (M = 4.20, SD = 0.88). In our regression model, practice-related characteristics, the expected future digital health usage, GPs’ digital affinity, several personality traits, and digital maturity are significant predictors of the perceived strength of barriers. For the perceived importance of improvement strategies, only demographics and usage-related variables are significant predictors. This study provides strong evidence for the impact of GPs’ inherent characteristics on barriers and improvement strategies. Our findings highlight the need for comprehensive approaches integrating personal and emotional elements to make digitization in practices more engaging, tangible, and applicable.

## Introduction

In the contemporary healthcare landscape, digital technologies have emerged as powerful tools, offering the potential to improve health outcomes^1,2^, reduce costs^3^, enhance patient care^4,5^, and improve the effectiveness and efficiency of healthcare delivery^3,6,7^. This spread of digital health solutions was further accelerated by the COVID-19 pandemic^8^. Despite the potential benefits of digital health solutions, their adoption and successful integration into healthcare organizations has been slow^9,10^ and impeded by various barriers^11,12^. As the digitalization of healthcare continues to reshape medical practices, understanding and addressing perceived barriers among general practitioners (GPs) is paramount. In this context, extensive research has studied digital health adoption across various medical disciplines, healthcare settings, and technologies, ranging from remote consultations^13,14^ to mHealth^15,16^, electronic medical records^17,18^, and remote monitoring^19,20^. Today, only a few studies considered a broader perspective on digital health adoption^21^, investigated potential strategies to improve adoption^22,23^, and studied potential influencing factors^24^.

Amid the digitalization of healthcare, GPs can choose various digital health solutions for their practice, ranging from video consultations and mobile health apps to digital appointment booking. As GPs are most patients’ primary point of contact^25^ in European healthcare systems, they are thus at the center of providing comprehensive and continuous healthcare services^26^. Consequently, GPs’ adoption and effective utilization of digital health solutions significantly impact the integration of these technologies into routine clinical practice^12^ and, hence, influence patient care. Moreover, GPs’ adoption of digital health solutions can enhance their job satisfaction and work-life balance^27^.

Therefore, understanding factors influencing the barriers perceived among GPs is vital to fostering the effective and sustainable adoption of digital health solutions in a rapidly evolving landscape. By digital health solutions, in this study, we mean digital tools, technologies, and services designed to improve healthcare, make it more efficient, and personalize it. This includes the use of digital services (e.g., video consultations, digital telephone assistance system, digital appointment booking, digital medical history, digital practice administration) and the use of connected medical devices and artificial intelligence (e.g., telemonitoring, decision support systems).

Through a mixed-methods research approach combining qualitative (i.e., literature review, expert interviews) and quantitative methodologies (i.e., online survey), we aim to identify adoption barriers and potential strategies for improvement in general practice settings more broadly and further evaluate their association with GPs’ inherent characteristics, especially their personality. As the research on influencing factors to digital health adoption in general practices is limited, we close this gap by providing a more nuanced understanding of inherent characteristics and their effect on digital health adoption among GPs. Understanding these inherent influencing factors enables the development of evidence-based, targeted strategies to address resistance and facilitate the successful integration of digital health solutions into clinical practice, whether through communication styles that resonate with different personality types or by providing additional support to individuals less comfortable with technological change. Tailoring interventions to the specific needs and characteristics of GPs enhances the effectiveness of digital health adoption strategies.

## Results

### Adoption barriers and improvement strategies in general practices (literature review and expert interview results)

Our literature review and expert interviews aimed to identify and synthesize currently postulated adoption barriers and improvement strategies for digital health adoption more broadly and validate their relevance in general practice settings. We initially retrieved 1276 records in the literature search, of which we included 24 articles^13,15,17–19,22,23,28–44^.

The literature review identified technological, social, and organizational adoption barriers. More than 90% of included studies reported organizational adoption barriers^13,15,17,18,22,23,28–44^ (23/24), with more than half reporting high workload^17,22,29,30,34,36–44^ and a lack of time^13,15,17,18,23,28,29,31–34,36,40,42^ (each 14/24; 58%) as predominant barriers. Another 88% of studies identified social adoption barriers^13,15,17,18,22,23,28–32,35–44^ (21/24). Of these, physicians’ familiarity with digital health solutions^15,17,18,22,23,28,30–32,36,38–44^ (17/24; 71%) was the most cited social barrier, followed by overall awareness^15,18,22,23,29,30,32,35,43,44^ (10/24; 42%) and patient preferences^15,18,23,29–31,35,38,40,42^ (10/24; 42%).

Our ten expert interviews with GPs confirmed and validated the relevance of all three categories of barriers and five categories of improvement strategy. Overall, the relevance of the three categories of barriers was consistently rated as high. In line with the high estimated relevance, all GPs mentioned technological barriers, especially regarding system reliability (10/10; 100%), usefulness (9/10; 90%), and technical support (9/10; 90%). Additionally, most GPs mentioned the familiarity and ability of practice staff (each 8/10; 80%), patients’ preferences and ability (8/10; 80%), a lack of reimbursement (9/10; 90%), a high workload and lack of time (each 9/10; 90%), and the socio-political context (9/10; 90%) as substantial adoption barriers. On average, GPs reported around 14 different barriers.

Looking into potential strategies to support and improve digital health adoption, we identified strategies in five categories in our literature review: development-related, awareness-related, knowledge-related, implementation-related, and policy-related strategies. Around two-thirds of studies identified strategies concerning the development of digital health solutions as potentially helpful to improve adoption^15,17–19,28,29,31,33,34,36–39,42–44^ (16/24; 67%). Among these, the most frequently cited development-related strategies were improvements in the usefulness of digital health solutions^17–19,28,29,31,33,36–38,42–44^ (13/24; 54%), followed by improvements in their usability^28,29,31,34,36,39,42,43^ (8/24; 33%). All other categories were present in around half of the included studies, with the call for ongoing training^15,17–19,34,37,38,40,43,44^ (10/24; 42%) and improved reimbursement^15,17,18,22,34,38,39,43^ (8/24; 33%) as additionally vital improvement strategies.

**Fig. 1:**
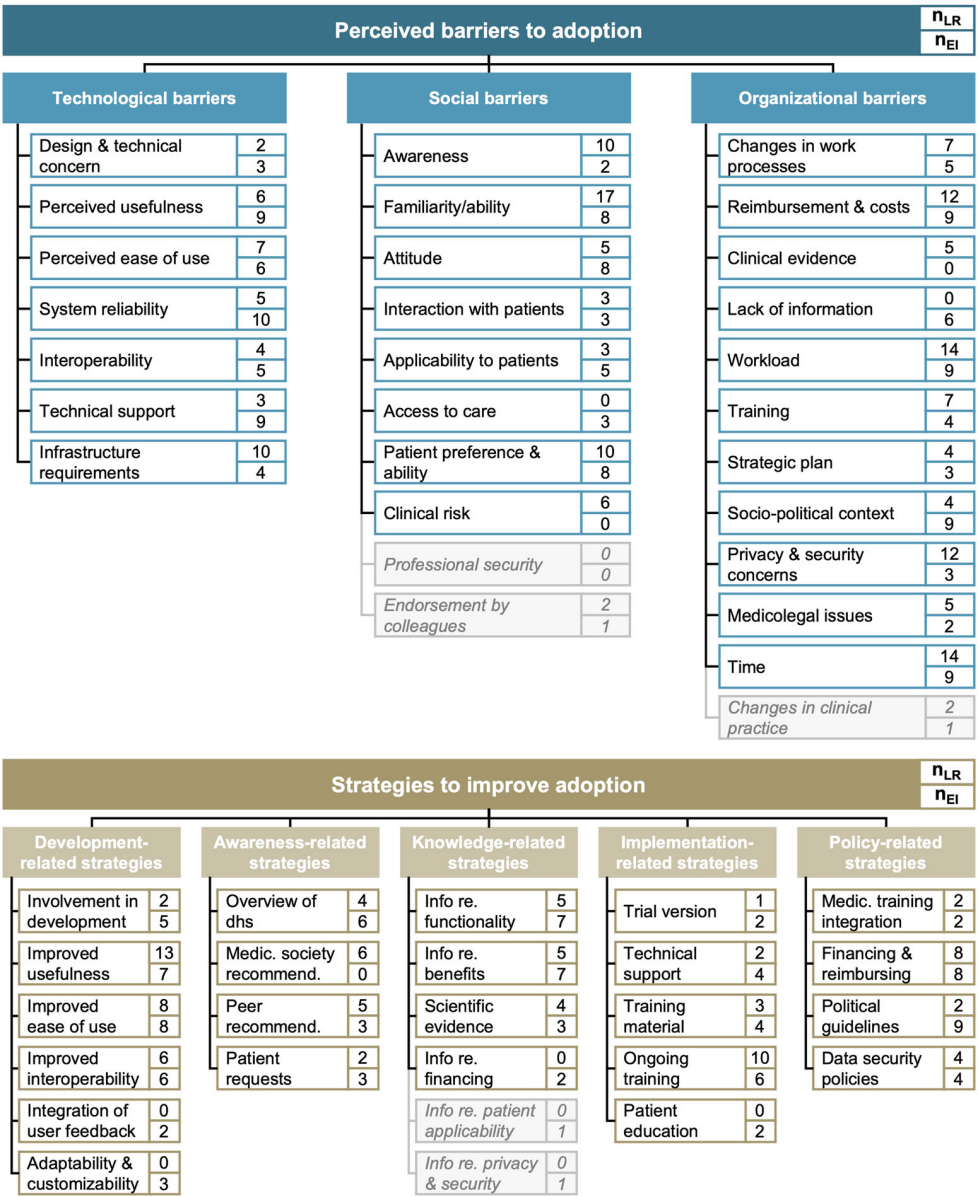
Overview of categories and individual barriers (strategies) based on the literature review and expert interview results. The figure shows categories and corresponding individual barriers (strategies) as well as their appearance in the literature review and expert interviews. n_LR_ represents the number of studies identified in the literature review proposing the barrier (strategy); n_EI_ shows the number of expert interviews in which the barrier (strategy) was mentioned. Light grey boxes with italic text show barriers (strategies) not included in the subsequent online survey. dhs digital health solutions.

Our expert interviews further highlighted that GPs considered development-related strategies particularly relevant: 80% of GPs would like to see improved usability of digital health solutions (8/10). In addition, GPs especially called for improvements in remuneration (8/10; 80%) and a simplification of political guidelines (9/10; 90%). Awareness-related strategies were rated as least relevant (6.2/10.0). Of these, GPs wished for further information on the functionalities and benefits of digital health solutions (each 7/10; 70%). Overall, GPs reported around 11 strategies. In our subsequent online survey, we only included items for barriers or strategies proposed by more than four articles or mentioned by more than one interviewee to ensure theoretical and expert consensus. Figure 1 shows the synthesized results.

### Factors influencing adoption barriers and improvement strategies (online survey results)

To analyze factors that may influence adoption barriers and improvement strategies, our online survey focused on five areas of inherent characteristics: (i) demographics and practice-related characteristics, (ii) digital health usage, (iii) digital affinity, (iv) personality, and (v) digital maturity of the practice.

**Fig. 2:**
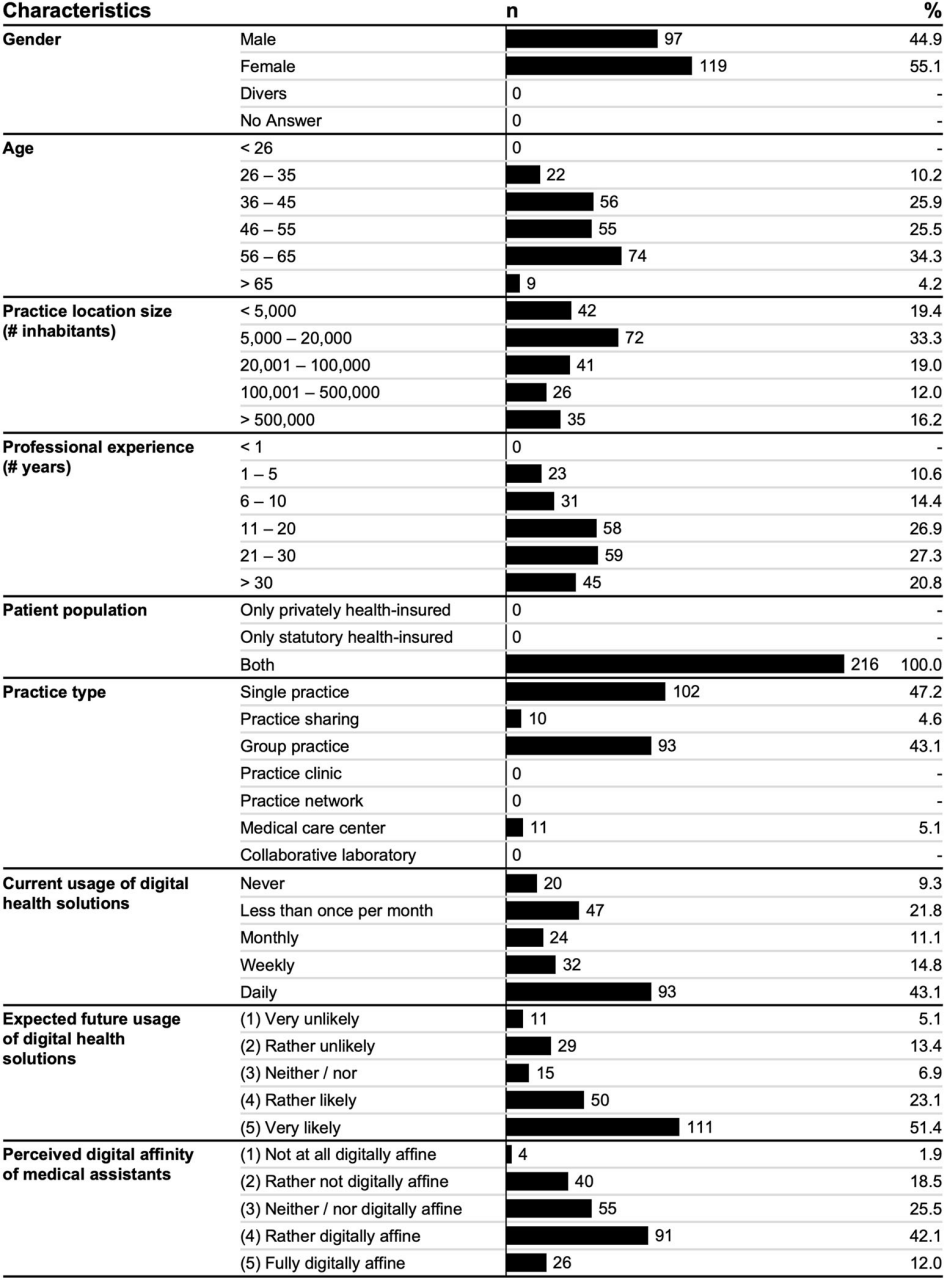
Characteristics of participating GPs (*N* = 216). The figure shows assessed individual and practice-related characteristics of participating GPs.

After data cleaning, quality, and privacy control, we included a broad sample of 216 German GPs with a diverse set of demographics (see Fig. 2).

Around half of respondents used digital health solutions daily (93/216; 43.1%), while almost a third did not use them at all (20/216; 9.3%) or rather seldomly (47/216; 21.8%). Most respondents further expected to rather or very likely use digital health solutions in the future (161/216; 74.5%).

Further, GPs perceived the work-related digital affinity of their medical assistants to be moderate (55/216; 25.5%) or relatively high (91/216; 42.1%) and had a relatively moderate affinity for technology interaction^45^ themselves (M = 2.66, SD = 1.08).

Concerning personality^46^, respondents can be characterized as highly conscientious (M = 4.10, SD = 0.59) and open (M = 3.85, SD = 0.68), moderately extroverted (M = 3.64, SD = 0.80) and agreeable (M = 3.53, SD = 0.75), and mildly neurotic (M = 2.42, SD = 0.71). The digital maturity of their practices was moderate (M = 3.32, SD = 0.64).

**Fig. 3:**
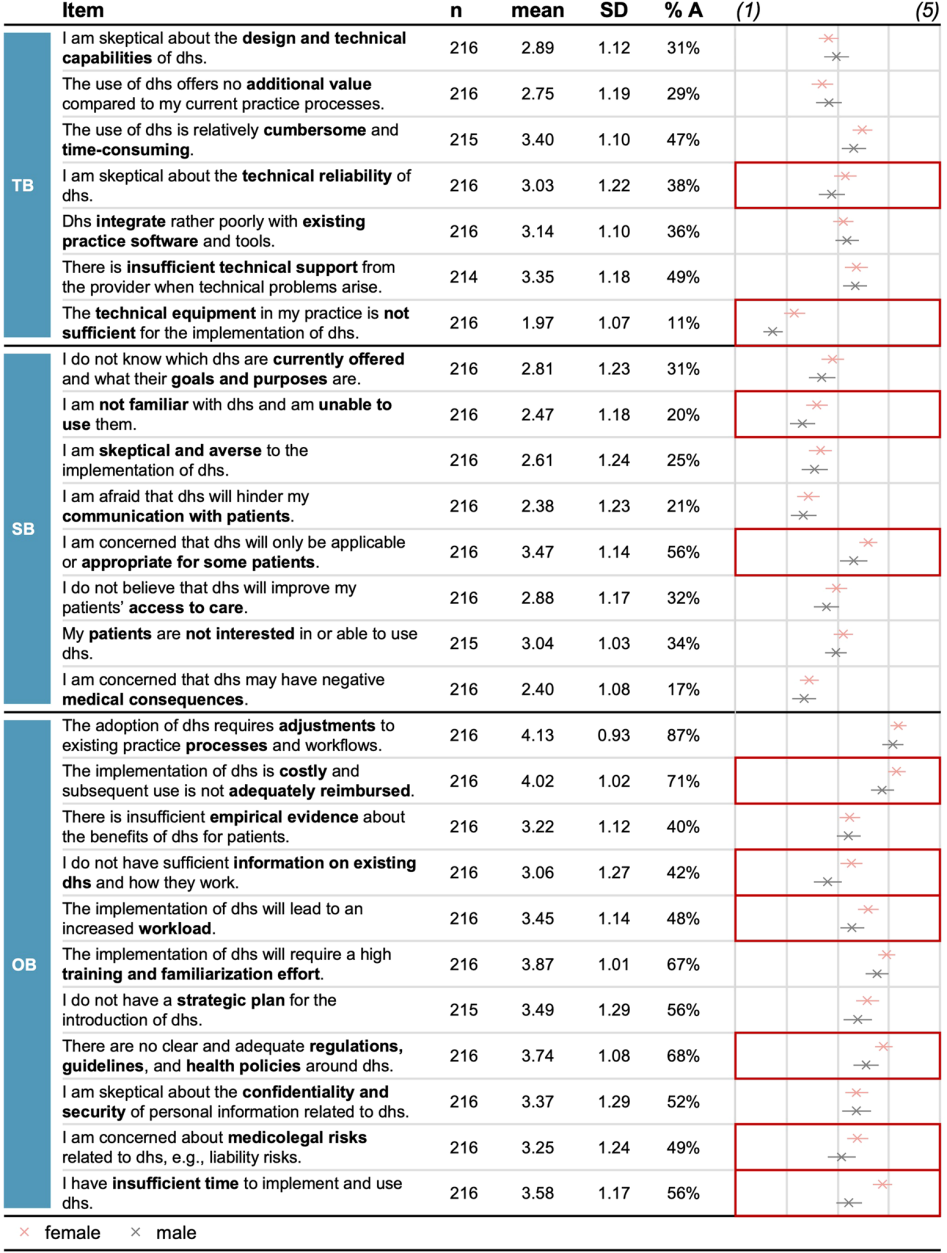
Sample size, mean, standard deviation, agreement rates, and between-group comparison for adoption barriers along the categories assessed. The figure shows items for adoption barriers per category, the respective sample size, descriptive statistics, and between-group comparison. The dot chart shows the mean value per item. Error bars represent +/− 2 standard errors. Cells with red framing show substantial differences between groups. %A Percentage of respondents agreeing to the statements and thus rating the respective barrier as relevant rating of (4) or (5); TB technological barriers; SB social barriers; OB organizational barriers; dhs digital health solutions.

Overall, respondents saw around 11 barriers (M = 11.12, SD = 6.01) and rated these as relatively moderate (M = 3.08, SD = 0.68). Among the three categories, organizational barriers were rated highest on average (M = 3.56, SD = 0.71), followed by technological (M = 2.93, SD = 0.76) and social (M = 2.76, SD = 0.79) barriers. For most individual barriers, scores were again moderate, with the highest rating for required workflow adjustments (M = 4.13, SD = 0.93), high costs and inadequate reimbursement (M = 4.02, SD = 1.02), and a high training and familiarization effort (M = 3.87, SD = 1.01) as the top three barriers (see Fig. 3).

**Fig. 4:**
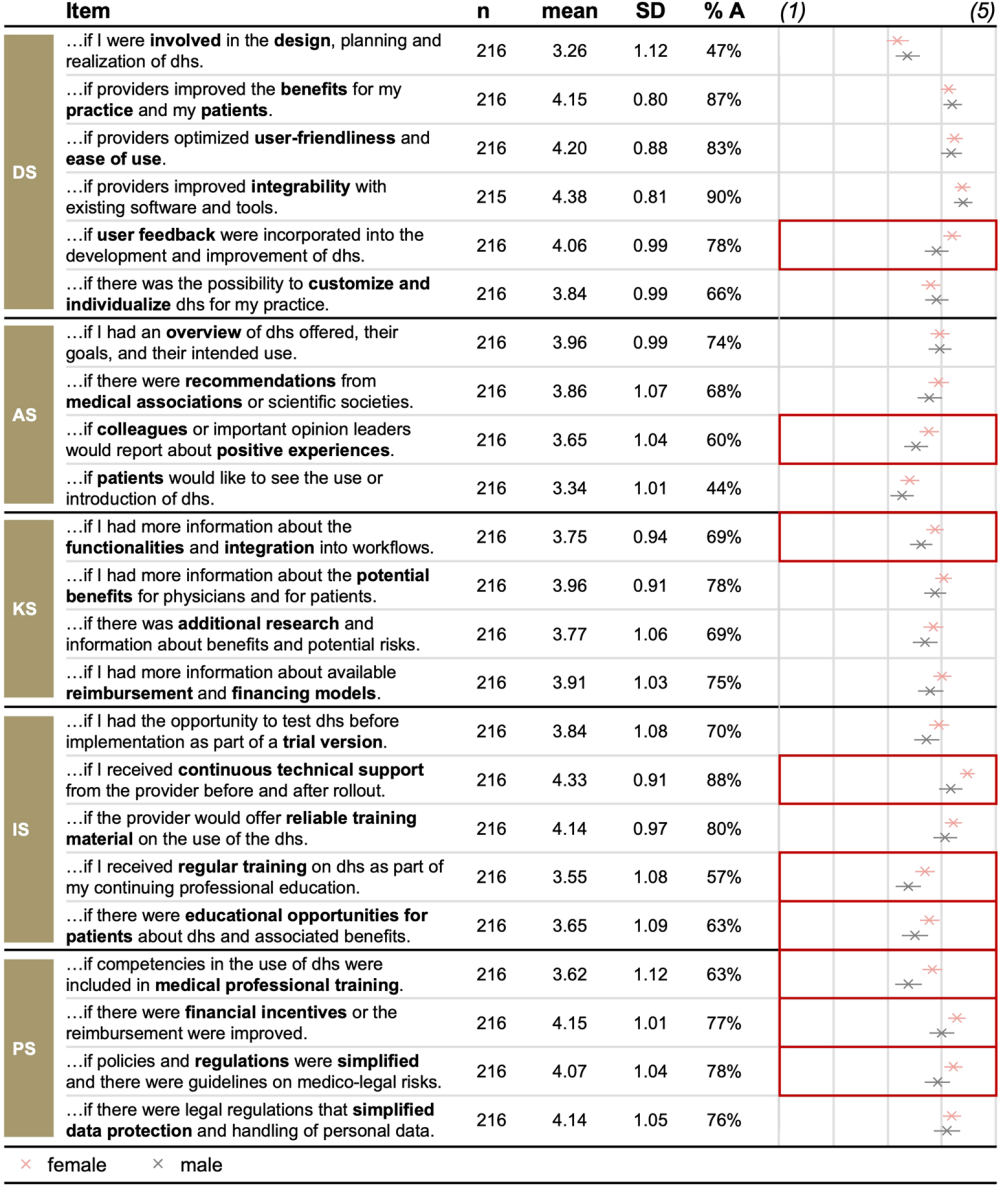
Sample size, mean, standard deviation, agreement rates, and between-group comparisons for improvement strategies along the categories assessed. The figure shows items for improvement strategies per category, the respective sample size, descriptive statistics, and between-group comparisons. The dot chart shows the mean value per item. Error bars represent +/−2 standard errors. Cells with red framing show substantial differences between groups. %A Percentage of respondents agreeing to the statement and thus rating the respective strategy as important rating of (4) or (5); DS development-related strategies; AS awareness-related strategies; KS knowledge-related strategies; IS implementation-related strategies; PS policy-related strategies; dhs digital health solutions.

On average, respondents perceived around 16 improvement strategies as important (M = 3.89, SD = 0.61). Policy-related (M = 4.00, SD = 0.81) and development-related strategies (M = 3.98, SD = 0.67) received the highest rating, followed by implementation-related (M = 3.90, SD = 0.78) and knowledge-related strategies (M = 3.85, SD = 0.81). Awareness-related strategies scored lowest but were also perceived as important (M = 3.70, SD = 0.74). Most individual strategies were similarly rated important (see Fig. 4): Respondents especially wished for improved interoperability (M = 4.38, SD = 0.81), continued technical support (M = 4.33, SD = 0.91), and improved usability (M = 4.20, SD = 0.88).

**Fig. 5:**
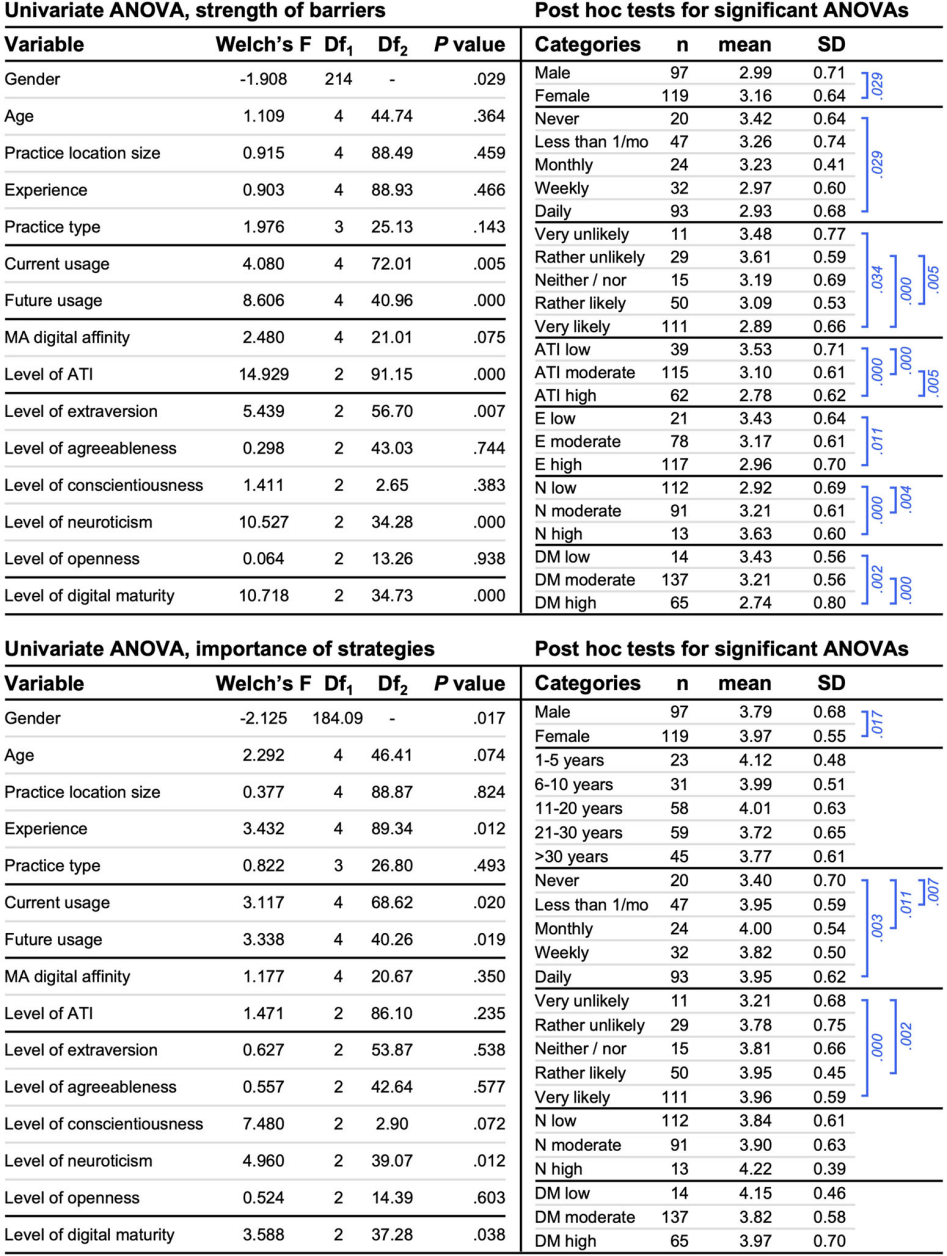
Univariate ANOVAs and post hoc tests. Both parts of the figure show the results for Welch ANOVAs (left) and Hochberg GT2 or Games-Howell post hoc tests for significant Welch ANOVAs in the order of appearance (right). The upper part reports results for the strength of barriers, the lower part reports results for the importance of strategies. Blue brackets represent significant comparisons. As gender is a dichotomous variable, we conducted a two-tailed *t*-test. The results show the *t*-statistic (in the column ‘Welch’s F’), its’ df, and *P*-value. MA digital affinity medical assistants’ digital affinity; ATI affinity for technology interaction; N neuroticism; DM digital maturity.

We conducted separate univariate ANOVAs and post hoc tests, to assess differences in the number and strength of adoption barriers and the number and importance of improvement strategies given the several inherent characteristics considered (see Fig. 5).

The strength of barriers differed based on gender, current and future use of digital health solutions, GPs’ level of affinity for technology interaction, the level of extraversion and neuroticism, and the level of digital maturity. Post hoc tests revealed that participants who were female (M = 3.16, SD = 0.64, *Cohen’s d* = 0.25), never used digital health solutions (M = 3.42, SD = 0.64, *p* = 0.029, *Cohen’s d* = 0.73; Hochberg GT2 post hoc test), were very (M = 3.48, SD = 0.77, *p* = 0.034, *Cohen’s d* = 0.88; Hochberg GT2 post hoc test) or rather unlikely to use digital health solutions in the future (M = 3.61, SD = 0.59, *p* < 0.001, *Cohen’s d* = 1.12; Hochberg GT2 post hoc test), had a low level of affinity for technology interaction (M = 3.53, SD = 0.71), a low level of extraversion (M = 3.43, SD = 0.64, *p* = 0.011, *Cohen’s d* = 0.68; Hochberg GT2 post hoc test), a high (M = 3.63, SD = 0.60, *p* < 0.001, *Cohen’s d* = 1.04; Hochberg GT2 post hoc test) or moderate level of neuroticism (M = 3.21, SD = 0.61, *p* = .004, *Cohen’s d* = 0.44; Hochberg GT2 post hoc test), and a low (M = 3.43, SD = 0.56, *p* = 0.002, *Cohen’s d* = 0.90; Games-Howell post hoc test) or moderate level of digital maturity (M = 3.21, SD = 0.56, *p* < 0.001, *Cohen’s d* = 0.73; Games–Howell post hoc test) reported a higher strength of barriers compared to respondents who were male, used digital health solutions daily, were rather or very likely to use digital health solutions in the future, had a moderate or high level of affinity for technology interaction, a high level of extraversion, a low level of neuroticism, or a high level of digital maturity. Interestingly, male and female participants rated poor compatibility with work processes, a lack of reimbursement, high costs, and a high training effort as the most substantial adoption barriers (see Fig. 3).

We found a similar pattern for the number of barriers, except that there was no difference between GPs based on gender (*t*(214) = −1.397, *p* = 0.082; *t*-test), yet a significant difference based on the perceived digital affinity of medical assistants (Welch’s *F*(4, 21.51) = 3.433, *p* = .003; Welch ANOVA). GPs who perceived their medical assistants to be somewhat not digitally affine (M = 13.60, SD = 5.13) reported significantly more adoption barriers adoption compared to respondents perceiving their medical assistants to be fully digitally affine (M = 9.42, SD = 5.87, *p* = 0.053, *Cohen’s d* = 0.77; Hochberg GT2 post hoc test).

Looking at the importance of improvement strategies, we found significant differences between GPs based on gender, professional experience, current usage and expected future digital health usage, the level of neuroticism, and the level of digital maturity (see Fig. 5). Post hoc tests revealed that respondents who were female (M = 3.97, SD = 0.55, *p* = 0.017, *Cohen’s d* = 0.29; *t*-test), used digital health solutions daily (M = 3.95, SD = 0.62, *p* = 0.003, *Cohen’s d* = 0.87; Hochberg GT2 post hoc test), monthly (M = 4.00, SD = 0.54, *p* = 0.011, *Cohen’s d* = 0.97; Hochberg GT2 post hoc test), or seldomly (M = 3.95, SD = 0.59, *p* = .007, *Cohen’s d* = 0.88; Hochberg GT2 post hoc test), and were very (M = 3.96, SD = 0.59, *p* < 0.001, *Cohen’s d* = 1.25; Hochberg GT2 post hoc test) or rather likely to use digital health solutions in the future (M = 3.95, SD = 0.45, *p* = 0.002, *Cohen’s d* = 1.49; Hochberg GT2 post hoc test), reported a higher importance of strategies, compared to respondents who were male, never used digital health solutions, and were very unlikely to use these in the future. Interestingly, female participants viewed continuous technical support, improved interoperability, and improved reimbursement as the most vital improvement strategies. In contrast, for male participants, it was an enhanced interoperability, improved usefulness, and improved usability (see Fig. 4). Overall, we found similar results for the number of improvement strategies, except that there was an additional significant difference based on respondents’ level of conscientiousness (Welch’s *F*(2, 3.34) = 11.988, *p* = 0.030; Welch ANOVA).

In the next step, we conducted a linear hierarchical regression analysis to deepen our understanding of the association between adoption barriers, improvement strategies, and GPs’ inherent characteristics.

Looking at adoption barriers (see Table 1), demographics, practice-related characteristics, and digital health usage alone explained about 21.8% of the variance in the strength of barriers, reaching statistical significance of the model, *F*(21, 194) = 2.573, *p* < 0.001 (F-test). When including digital affinity variables in model 3, the proportion of explained criterion variance increase by 8.8% to an overall *R*^*2*^ of around 30.6% (*F*(23, 192) = 3.684, *p* < 0.001; F-test). Further including personality traits into our model led to an additional increase in *R*^*2*^ of 10.3%. Finally, also including digital maturity led to an increase in *R*^*2*^ of 3.6% to an overall *R*^*2*^ of 44.5% (*F*(29, 186) = 5.139, *p* < 0.001; F-test). Thus, our model significantly improved at each stage of the hierarchical process. The same was true for the number of adoption barriers, with a final *R*^*2*^ of 42.6% (*F*(29, 186) = 4.762, *p* = 0.005; F-test).

**Table 1:** Model parameters of the linear hierarchical regression model for the strength of barriers

Model #	Included variables	F	Df_M_	Df_R_	*P* value	R^2^	ΔR^2^	Δ *P* value
1	Demographics & practice-related characteristics	1.198	16	199	0.272	0.088	0.088	0.272
2	Demographics & practice-related characteristics + Digital health usage	2.573	21	194	<0.001	0.218	0.130	<0.001
3	Demographics & practice-related characteristics + Digital health usage + Digital affinity	3.684	23	192	<0.001	0.306	0.088	<0.001
4	Demographics & practice-related characteristics + Digital health usage + Digital affinity + Personality	4.620	28	187	<0.001	0.409	0.103	<0.001
5	Demographics & practice-related characteristics + Digital health usage + Digital affinity + Personality + Digital maturity	5.139	29	186	<0.001	0.445	0.036	<0.001

The table provides an overview of model parameters for the linear hierarchical regression model. *P*-values refer to the results of the corresponding F-test. Variables included in the different stages of the hierarchical approach are as follows: Model 1 (gender, age dummy coded, practice location size dummy coded, experience dummy coded, practice type dummy coded); Model 2 (model 1 variables, current usage dummy coded, expected future usage); Model 3 (model 2 variables, perceived digital affinity of medical assistants, GPs’ affinity for technology interaction); Model 4 (model 3 variables, extraversion, agreeableness, conscientiousness, neuroticism, openness); Model 5 (model 4 variables, digital maturity).

In our final regression model, eight variables were significantly associated with the strength of barriers (see Supplementary Table 1 and Supplementary Table 2 for a detailed overview of coefficients). The strength of barriers was significantly associated with the practice location, the practice type, the expected future use of digital health solutions, GPs’ affinity for technology interaction, their extraversion, neuroticism, and openness, and the digital maturity of the practice. Accordingly, practicing in cities with less than 5,000 inhabitants compared to cities with 100,001 to 500,000 inhabitants (*b* = −0.315, *SE B* = 0.142, *β* = −0.152, *p* = 0.028; *t*-test) or cities with more than 500,000 inhabitants (*b* = −0.301, *SE B* = 0.133, *β* = −0.164, *p* = 0.025; *t*-test), sharing practices (*b* = 0.498, *SE B* = 0.189, *β* = 0.155, *p* = 0.009; *t*-test) compared to single practices, a lower expected likelihood of future usage (*b* = −0.151, *SE B* = 0.051, *β* = −0.281, *p* = 0.003; *t*-test), a lower affinity for technology interaction (*b* = −0.159, *SE B* = 0.042, *β* = −0.254, *p* < 0.001; *t*-test), lower extraversion (*b* = −0.109, *SE B* = 0.055, *β* = −0.129, *p* = 0.048; *t*-test), higher neuroticism (*b* = 0.156, *SE B* = 0.063, *β* = 0.164, *p* = 0.014; *t*-test) and openness (*b* = 0.134, *SE B* = 0.062, *β* = 0.135, *p* = 0.031; *t*-test), and lower digital maturity (*b* = −0.247, *SE B* = 0.071, *β* = −0.236, *p* < 0.001; *t*-test) were associated with a higher strength of barriers. We found similar results for the linear hierarchical regression model predicting the number of barriers, except that there was no substantial association with the practice type or extraversion.

Looking at the importance of improvement strategies (see Table 2), the model only including demographics and practice-related characteristics explained about 10.2% of the variance but did not reach statistical significance (*F*(16, 199) = 1.407, *p* = 0.141; F-test). Including digital health usage in our model yielded significant improvement in the proportion of explained criterion variance by 9.8%, leading to a total *R*^*2*^ of 20.0% (*F*(21, 194) = 2.305, *p* = 0.002; F-test). Further including digital affinity, personality, or digital maturity as predictors did not significantly improve the model, although the respective regression models were significant (see Table 2). Thus, the regression model only including demographics, practice-related characteristics, and digital health usage best fit our data.

**Table 2:** Model parameters of the linear hierarchical regression model for the importance of improvement strategies

Model #	Included variables	F	Df_M_	Df_R_	*P* value	R^2^	ΔR^2^	Δ *P* value
1	Demographics & practice-related characteristics	1.407	18	199	0.141	0.102	0.102	0.141
2	Demographics & practice-related characteristics + Digital health usage	2.305	21	194	0.002	0.200	0.098	<0.001
3	Demographics & practice-related characteristics + Digital health usage + Digital affinity	2.109	23	192	0.003	0.202	0.002	0.788
4	Demographics & practice-related characteristics + Digital health usage + Digital affinity + Personality	1.829	28	187	0.010	0.215	0.013	0.675
5	Demographics & practice-related characteristics + Digital health usage + Digital affinity + Personality + Digital maturity	1.757	29	186	0.014	0.215	0.000	0.874

The table provides an overview of model parameters for the linear hierarchical regression model. *P*-values refer to the results of the corresponding F-test. Variables included in the different stages of the hierarchical approach are as follows: Model 1 (gender, age dummy coded, practice location size dummy coded, experience dummy coded, practice type dummy coded); Model 2 (model 1 variables, current usage dummy coded, expected future usage); Model 3 (model 2 variables, perceived digital affinity of medical assistants, GPs’ affinity for technology interaction); Model 4 (model 3 variables, extraversion, agreeableness, conscientiousness, neuroticism, openness); Model 5 (model 4 variables, digital maturity).

In this model (model 2), three variables were significantly associated with the importance of improvement strategies. We found a significant association with respondents’ professional experience, their current usage of digital health solutions, and their expected future usage. Having 1 to 5 years of professional experience compared to 21 to 30 (*b* = −0.524, *SE B* = 0.210, *β* = −0.381, *p* = 0.013; *t*-test), using digital health solutions seldom (*b* = 0.458, *SE B* = 0.165, *β* = 0.308, *p* = 0.006; *t*-test) or monthly (*b* = 0.430, *SE B* = 0.208, *β* = 0.221, *p* = 0.040; *t*-test) compared to never, and a higher expected likelihood of future usage (*b* = 0.105, *SE B* = 0.052, *β* = 0.216, *p* = 0.043; *t*-test) were associated with a higher importance. Again, results were similar for the linear hierarchical regression model of the number of improvement strategies, except that there was an additional significant association with respondents’ age: Being aged between 46 and 55 (*b* = 3.682, *SE B* = 1.720, *β* = 0.302, *p* = 0.034; *t*-test) or older than 65 (*b* = 6.218, *SE B* = 2.849, *β* = .234, *p* = 0.030; *t*-test) was significantly associated with a higher number of strategies.

## Discussion

Despite the high potential value of digital health solutions^1–3,6,7^, broad adoption and successful integration into healthcare organizations have been challenging^9,10^. This study systematically examined the influence of GPs’ personal and practice characteristics on adoption barriers and strategies to improve digital health adoption. In a linear hierarchical regression model, practice-related characteristics, the expected future digital health usage, GPs’ digital affinity, several personality traits, and digital maturity were significant predictors of the perceived strength of barriers. For the perceived importance of improvement strategies, demographics, and digital health usage-related variables were again significant predictors.

In line with previous research^11,16,22^, respondents saw multiple adoption barriers and rated these as rather moderate in our study. A recent systematic review^11^ found that organizational adoption barriers were more prevalent than technological factors. Likewise, our study obtained the highest scores for organizational barriers, followed by technological and social barriers. This highlights the clear importance of organizational factors for digital health adoption that go beyond the pure technical features of the solutions themselves, contrasting with another previous review^16^. However, a comparison of both studies should be interpreted cautiously, given the rapidly evolving digital health landscape and technical improvements in tools and services accelerated by the COVID-19 pandemic^8^.

The main barriers identified in our study include poor compatibility with work processes, insufficient reimbursement and high costs, a required high training and familiarization effort, inadequate and indistinct regulations, guidelines, healthcare policies, and a workload-related lack of time. Most of these barriers are consistent with those identified in previous literature^11,16,17,22^. A recent systematic review^17^ identified several influencing factors to adoption, highlighting a lack of interoperability that limits GPs’ ability to integrate digital health solutions flawlessly into existing workflows and exchange information with other healthcare providers as a strong barrier.

It is further not surprising that the high costs for implementing digital health solutions and a lack of reimbursement of corresponding services were proposed as core barriers in our study. Likewise, GPs mentioned financial incentives for digital health adoption as one of the most important improvement strategies. In line with this, a study on economic influencing factors for the acceptance of remote monitoring in Germany reported missing reimbursement arrangements, uncertain economic advantages, and missing business models as core barriers^20^. Interestingly, these findings contradict research highlighting the financial advantages of digital health solutions^3^ but might be explained by the relatively low usage of digital health solutions in our sample. As pointed out by previous studies^20^, current users saw substantially greater financial benefits than non-users. Our finding of usage-related differences further seconds this: GPs using digital health solutions daily perceived barriers overall to be lower compared to GPs never using these.

It is promising that only 21% of respondents in our survey were afraid that using digital health solutions would hinder their communication with patients. Previous studies reported a potential disruption during visits due to the use of mHealth^47^. This finding is in line with a more recent systematic review, showing the impact of digital health solutions on patient-professional interaction was more often deemed a facilitator of digital health adoption, as digital health solutions could facilitate the relationship with patients by providing a new means of communication^16^.

Interestingly, GPs perceived insufficient technical infrastructure as a minor adoption barrier in our study. This contrasts with the results of our literature review and previous studies reporting a poor information technology infrastructure as a constant barrier^15,31,42^. The findings might be attributable to geographic differences in digital health requirements. In Germany, the Act on Secure Digital Communication and Applications in the Healthcare System (‘Gesetz für sichere digitale Kommunikation und Anwendungen im Gesundheitswesen’)^48^ has established a legal framework for setting up the secure telematics infrastructure. Since then, various laws have advanced the digitalization of the German healthcare system, based on which, for example, general practices are required to use an electronic patient record (ePA), provide an electronic statement of fitness for work (eAU), and communicate via a uniform standard for the electronic transmission of healthcare-related documents (KIM). These standards have required practices to adopt a sufficient technical infrastructure to provide the services mentioned and providers to ensure the integrability of new digital health solutions.

To address these barriers and support the adoption of digital health solutions, GPs in our study explicitly wished for improvements in the interoperability of digital health solutions, continued technical support from providers, improvements in the usability and usefulness of digital health solutions, as well as financial incentives and simplifications in regulations for data protection. Most of these strategies are consistent with previous research^16,29,36^. Interestingly, a recent mixed-methods study on mHealth adoption in Germany^22^ found additional information to be the most important measure. As we assessed various knowledge-related strategies, our results provide clarity as to which types of content are most critical for GPs: Respondents in our study perceived information about the digital health solutions offered as most helpful, followed by information about potential benefits for themselves and their practices, information about available reimbursement and financing models, and scientific evidence. These findings further underline the call for more research on medical evidence of the benefits of digital health adoption^15,22,40^, which is subsequently presented in a structured and transparent way and made publicly available via various channels, including medical newspapers, magazines, or conferences.

While extensive research has studied influencing factors to digital health adoption^17,21,24,49^, no study has investigated factors influencing adoption barriers or improvement strategies. According to our findings, the strength of barriers and the importance of strategies differed between GPs based on gender, with female participants perceiving barriers as higher and strategies as more important than their male colleagues. This is in line with studies reporting that being male was associated with using digital health technology^27,50^ while being female was associated with lower digital health adoption^49^. Other studies found no gender-based differences for digital health adoption^24^ or even a higher usage for female participants^51^.

A similarly inconclusive pattern of results can be obtained for age and professional experience. In our study both variables were significant predictors of the importance of strategies. Yet, we did not find a substantial association between age or experience and the strength of barriers. In line with our mixed findings, previous evidence is similarly inconclusive: while some studies found older physicians to be more likely to use digital health technology^27^, others observed the opposite to be true^11,24,50,51^ and report that younger general practice staff with lower professional experience are more digitally competent and confident^21^. Given the mixed findings concerning age and gender^17^, there might, in fact, be no difference in digital health adoption based on gender or age at all, the effects be limited to certain digital health solutions only, or covariates substantially influencing the effects found.

Interestingly, practice location and practice type were significant predictors of the strength of adoption barriers, a finding that is consistent with previous studies^17,21^. A recent study on digital readiness in general practices found that rurality was associated with lower digital readiness^21^. Similarly, respondents practicing in urban areas perceived barriers to be significantly weaker in our study. This finding might be explained by the more pronounced population aging in rural than in urban areas^52^. As GPs in rural areas thus might have to deal with older populations, they might perceive digital health solutions to not be applicable to their patient populations. This is further underlined by studies proposing patients’ digital literacy as a key adoption barrier^15,31,35^, which is further consistent with our findings. Future research should therefore investigate measures to overcome this potential digital divide to support GPs in rural areas.

We further observed substantial differences in the number and strength of barriers as well as the number and importance of improvement strategies based on the current and expected future usage of digital health solutions. Countless studies have proposed a lack of experience and familiarity with digital health solutions to be a key barrier to adoption^11,16,17,23,36,38,41^, a finding that is further consistent with our literature review and expert interviews. As the expected future was as a strong predictor in our linear hierarchical regression model, it might be beneficial to provide GPs with information highlighting the importance of digital health solutions, the latest advancements, and outlooks in digital health. This is consistent with the perceived lack of information and need for further information highlighted in a recent study^22^ and further seconded by our findings that GPs perceive various information as helpful for supporting digital health adoption.

Looking at digital affinity, GPs’ overall affinity for technology interaction was comparable to the general public in Germany^45^. Our literature review further highlighted that GPs’ familiarity with technologies in general and their digital literacy were perceived as facilitators or a lack thereof as a strong barrier to digital health adoption^11,16,17^. As the affinity for technology interaction provides a first indication of the actual use of technical systems in everyday settings^45^, this highlights the importance of digital skills for GPs to enable the efficient use and management of these in routine clinical practice^53^.

Concerning personality, our study found mixed results: Extraversion, neuroticism and openness were significant predictors of the perceived strength of barriers, while the perceived importance of improvement strategies only differed based on GPs’ level of neuroticism. Previous studies investigated the relationship between personality and digital health adoption^27^. Yet, no study has investigated the association between GPs’ personality traits and barriers or improvement strategies. Although studies have linked personality to technology adoption in general^54^ and to patients’ continued app usage^55^ this association seems to be rather weak for clinicians^27^. The specific results found in our study can be explained by looking at the associated personality traits: Extraversion can be characterized as being talkative, energetic, assertive, outgoing, and enthusiastic^56^. As the adoption of digital health solutions is largely driven by GPs themselves, their attitude is strongly linked to digital health adoption^11,16^. Thus, it is plausible that GPs with low levels of extraversion perceived barriers to be substantially stronger compared to respondents with higher levels of extraversion, and that this also holds in our linear hierarchical regression model. Consistent across barriers and improvement strategies, we observed differences between GPs based on their level of neuroticism. This, again, is in line with the characteristics of insecurity, anxiousness, and hostility associated with being neurotic^56^, which might as well translate to higher barriers to digital health adoption.

Interestingly, we obtained substantial differences in barriers and improvement strategies based on the practice’s digital maturity level. Digital maturity is a multifaceted construct that describes the digital status of healthcare facilities across various technological and organizational dimensions compared against a theoretical endpoint of maturity within the current digital health landscape^57^. Digitally mature practices have evolved along different dimensions and achieved a higher digital status. Consequently, the association found in our study is plausible, as digitally mature practices perceive lower barriers to digital health adoption. This is also consistent with studies linking prior experience with digital health solutions to higher adoption^11,16^. As digital maturity was not a significant predictor of the perceived importance of improvement strategies, this provides critical practical insights. The various improvement strategies identified in our study can be applied to practices regardless of their digital maturity. However, they should be tailored to demographics and practice-related characteristics that proved to be significant predictors and to associated barriers.

Based on the findings discussed above, various practical implications for providers, regulators, policymakers, and other healthcare stakeholders can be derived to support GPs in their digitalization efforts.

As the poor compatibility of digital health solutions with existing practice processes and workflows was a core adoption barrier in our study, there is a strong need for improvements in digital health solutions’ design, that calls providers of digital health solutions into action. Providers should pay paramount attention to ensuring a smooth integration with existing software and tools, high user friendliness, and continued technical support before and after implementation. This is underlined by studies highlighting that the design of digital health solutions is central for promoting patient access to digital health solutions and fostering patient adherence^22,58^. To further act on this proposal, regulators and policymakers could consider incentivizing providers of digital health solutions to address these technological barriers.

Based on our findings, ongoing training could further be a potential lever to satisfy the information needs discussed earlier. As GPs work in a profession with a typically high workload that has been additionally strained by the COVID-19 pandemic^59^, digitalization-related topics, including training on digital health solutions, would need to be performed outside of practice hours and thus might be perceived as an add-on to the actual medical work. To overcome this discrepancy, regulators, policymakers, and other healthcare stakeholders could consider providing incentives for training, for instance, by continuing medical education certifications, and by starting to raise awareness of the potential benefits of digitalization early on among medical students. Trainings should be centered around delivering skills concerning technology use and especially focused on digital health solutions, their benefits for practices and patients, and outlooks into future advancements. These efforts should not only be part of separate and dedicated trainings around digital health solutions but rather included into medical trainings. Such an integrated approach could discuss the use of digital health solutions in dedicated medical use cases, for example, regarding diabetes or asthma treatment which included elements of telehealth and remote monitoring. This combination of medical and digital aspects of care could be a fruitful approach to medical training, that is more interesting, applicable and tangible for GPs to experience potential benefits of digital health usage. In addition, recognizing the influence of personality traits on perceived barriers, policymakers could consider personalizing training programs to cater to the diverse characteristics of GPs to further enhance the effectiveness of training initiatives.

With one in two GPs in our study considering a heavy workload and lack of time as barriers to implementing digital health solutions, dedicated support is required. Following the operating model of GPs in the UK, where digitalization in general practices is oftentimes managed and taken care of by dedicated practice managers^60^, healthcare stakeholders could offer programs to non-medical staff to become dedicated digitalization officers focused on managing digitalization-related topics and consequently supporting and relieving GPs^61^.

In addition, regulators and policymakers should reconsider current reimbursement schemes for services related to digital health solutions and provide detailed information on financing models as well as financial benefits resulting from digital health adoption. This would allow GPs to identify suitable financing options for themselves that align with the economic goals of their practice and, in turn, alleviate perceived barriers around reimbursements and costs.

Recognizing GPs’ strong wish for improved interoperability in our study, policymakers could invest in initiatives that promote seamless integration of digital health solutions. Enhancing interoperability can streamline information exchange and improve the overall efficiency of healthcare delivery even beyond individual practices.

As our study has identified several characteristics inherent to GPs as substantial predictors of perceived adoption barriers, future approaches to supporting GPs with the integration of digital health solutions into clinical practice strategies should be tailored to these characteristics. Healthcare stakeholders such as the Association of Statutory Health Insurance Physicians (“Kassenärztliche Vereinigung”) could, for example, consider dedicated campaigns for GPs practicing in rural areas or in shared practices as these were more likely to perceive adoption barriers. Additionally, it could be worthwhile to develop interventions linked to our findings concerning personality. As extraversion and openness were associated with lower perceived barriers, stakeholders could develop interventions that aim to evoke emotions related to these personality traits, for example by choosing a gamified approach or allowing GPs to picture how their practice might look like in the future. Given that neuroticism was associated with higher perceived barriers, interventions and information campaigns should further convey confidence and a feeling of trust in the digital transformation process.

Although our study reveals important findings, it comes with several limitations. First, it must be noted that our research on the association of GPs’ inherent characteristics, barriers and strategies was exploratory and novice. This exploratory approach helps to identify key factors influencing digital health adoption among GPs and thus provides a foundation for future research endeavors. Although our study explores several potentially relevant variables in the context of digital health adoption in general practices, it may not exhaustively explore all relevant variables or factors influencing the phenomena. Similarly, we did not capture all adoption barriers or potential supporting measures identified in previous reviews^11,16^. As we based our assessment on a thorough literature review and expert interviews, we are confident that we covered a broad spectrum of relevant adoption barriers and potential improvement strategies in general practice settings. Compared to the single technology focus in previous studies^11^, our more comprehensive focus on digital health solutions allows us to draw a holistic picture while still being economical. Nevertheless, the findings should be replicated in future research to establish the robustness of our results.

Second, as there is a lack of evidence linking digital health adoption to healthcare quality^62^, overcoming the barriers and applying the strategies identified in our study does not necessarily lead to a higher quality of care. Although we assess adoption barriers and improvement strategies, we do not provide guidance on improving healthcare quality but rather highlight measures that healthcare stakeholders can utilize to support digitalization in general practice settings.

Third, as we focused on GPs in Germany, the results obtained might not translate to different geographies and healthcare systems. As our study’s findings align with previous literature across various countries^11,16,17^ and the role of GPs is similar across European countries^26^, the results can be applied to European healthcare systems. However, we cannot claim validity in other countries with different healthcare systems. Future research could take a cross-country approach to validate our findings and uncover differences in barriers and improvement strategies based on geography.

Fourth, the main results of our study stem from an online survey. This might be associated with a bias towards a population with a higher electronic literacy, as is typical for web-based research. While the affinity for technology interaction in our sample was relatively moderate and comparable to a quota sample from the general public in larger German cities^45^, we are confident that a selection bias does not skew the results of our study. Nevertheless, our approach might have resulted in a tendency towards GPs with a higher interest in digital health-related topics or especially interested in voicing their wishes regarding digitalization in general practice settings.

Lastly, we asked GPs to self-assess their affinity for technology interaction and personality in our online survey. While the self-assessment utilized provides an economical and practical approach to capture GPs’ inherent traits, such self-assessment might be influenced by cognitive biases or social desirability, potentially limiting the reliability of the assessment. As the affinity for technology interaction was comparable to the German population^45^ and we found a high Cronbach’s Alpha for the affinity for technology interaction in our study, we are confident that the results obtained are accurate. In addition, a recent study analyzing the psychometric properties of the scale used in our study highlights the reliability of the scale among several indices^63^. However, a longitudinal assessment would be needed to provide further confidence in the reliability of the assessment over time. Concerning personality, the scale used for the self-assessment of GPs’ personality traits in our study has already been shown to be relatively stable over time^46^, highlighting the reliability of the assessment.

By investigating various factors influencing adoption barriers and strategies for improvement, this study provides valuable insights into the personal, professional, and practice-related characteristics associated with the adoption of digital health solutions in general practice settings. GPs especially perceived organizational adoption barriers around poor workflow integrability, lack of reimbursement, and a high familiarization effort. We found practice-related characteristics, the expected future digital health usage, digital literacy, personality, and digital maturity being substantial predictors. To address these barriers and support the adoption of digital health solutions, GPs wish for several improvement strategies, especially concerning improved integrability and usability, technical support, and reliable training material. In conclusion, our findings highlight the need for approaches that not only cover pure information on digital health solutions but are integrated with more personal and emotional elements targeting the different inherent characteristics of GPs and, thus, making digitalization in practices more exciting, tangible, and applicable.

## Methods

### Study design

Data gathering and analysis for this study followed a mixed-methods approach using qualitative and quantitative methodologies. To identify relevant adoption barriers and improvement strategies in general practices, we first carried out a literature review in accordance with the PRISMA-ScR guidelines^64^. To validate the findings of our literature review and ensure their applicability to digital health solutions more broadly, we next conducted expert interviews with GPs based on the COREQ checklist^65^. Next, we created an online survey in accordance with the CHERRIES guideline^66^ for internet surveys to assess adoption barriers, improvement strategies, and relevant characteristics inherent to GPs, and ultimately answer the following research question:

Which personal and practice-related characteristics, usage-related factors, and personality traits substantially influence adoption barriers and improvement strategies?

All steps of this research project were approved by the Ethics Committee of Witten/Herdecke University (Nr. S-242/2022).

### Literature review

For our literature review, we followed the PRISMA-ScR guideline^64^ and searched the PubMed and PsycINFO databases accordingly (see Supplementary Table 3). To identify potentially relevant citations in both databases, we developed a search string covering three categories of keywords combined with the Boolean OR operator: (1) adoption, (2) digital health, (3) barriers/improvement strategies. For a more targeted view of digital health adoption in general practices, we added MeSH terms concerning GPs to our search (details are provided in Supplementary Table 3).

**Fig. 6:**
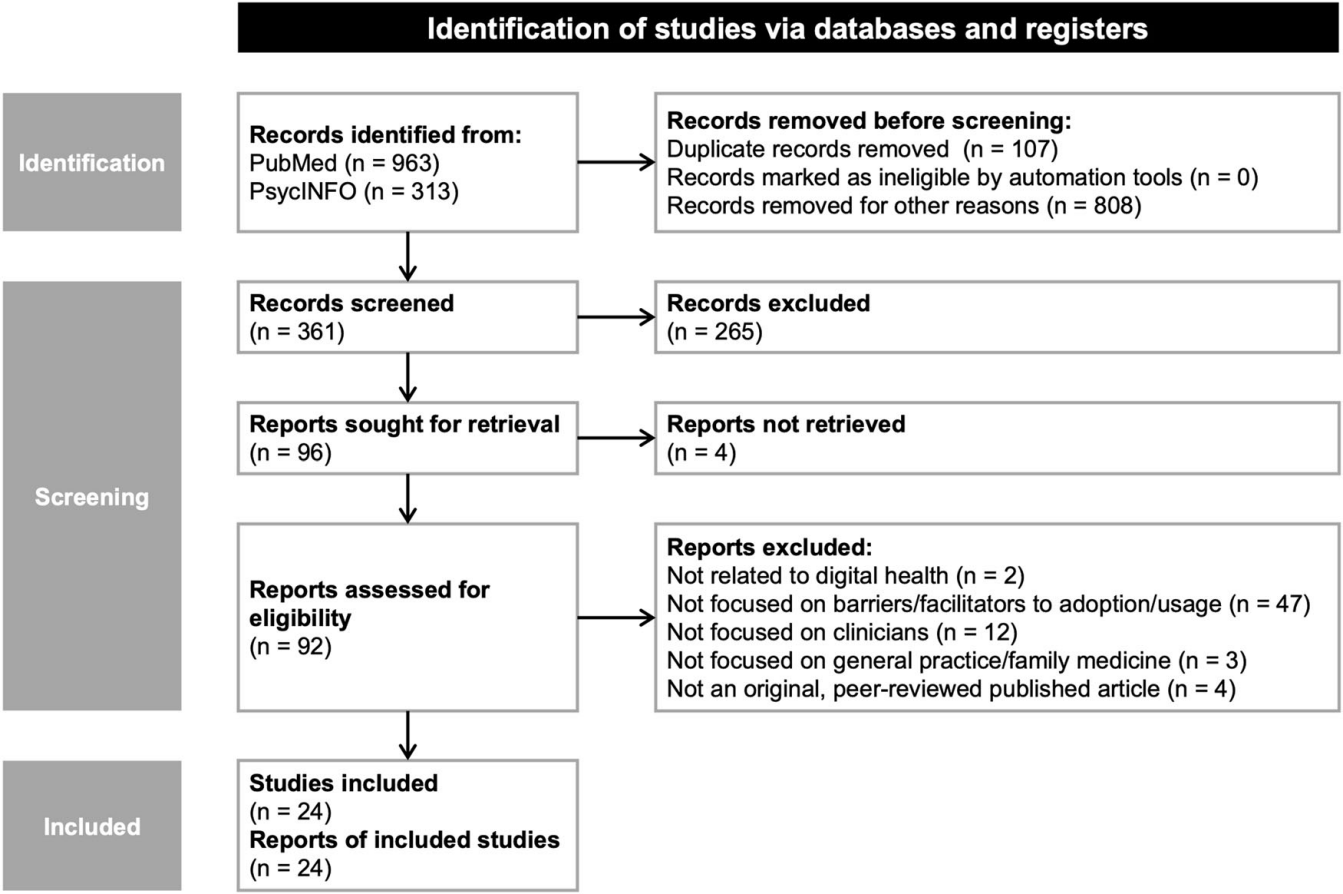
Flowchart for the literature review following PRISMA-ScR guidelines. The flowchart shows the sequential screening process during the literature review. ‘Records removed for other reasons’ shows records removed based on language and publication date criteria.

We initially retrieved 1276 citations from the two databases. After removing duplicates, we narrowed our search to more recent articles published between 2018 and 2022 in either English or German. As the COVID-19 pandemic has accelerated the adoption of digital health, this approach aimed at capturing more recent evolvements. For the remaining citations, we carried out abstract screening in accordance with our pre-defined inclusion criteria, resulting in 96 potentially relevant articles being retained. To determine eligibility, we further conducted a full-text review based on our inclusion criteria, leading to 24 papers being included in the review after screening (see Fig. 6 for the detailed screening process). Following our inclusion criteria, we selected the 24 articles as they focused on clinician populations, digital health solutions, and general practice settings and addressed, measured, or reported factors impacting or promoting the adoption or use of digital health solutions.

For the abstract and full-text screening, we excluded articles if they (1) were not related to digital health to maintain the focus on digital health and ensure the relevance for our research question; (2) did not address barriers or improvement strategies to digital health adoption or usage (i.e., focused on general attitudes, experiences, or the development or evaluation of digital health) to narrow the scope of our study; (3) were majorly focused on non-clinician populations (i.e., nurses or patients) as GPs hold a pivotal role in the digitalization of general practices and, thus, capturing their perspectives is of utmost importance; (4) were focused on care areas other than general practice as we believe that decision processes for adopting digital health solutions and subsequently implementing these essentially differ between different healthcare settings; and (5) were not original, peer-reviewed, published full-text articles to ensure the reliability and quality of the included literature. All inclusion and exclusion criteria used in this study were aligned in an expert panel before screening. Evidence from the included studies was synthesized by extracting and grouping potentially relevant barriers per a framework proposed in a recent review^11^ and improvement strategies based on the underlying adoption process steps. Thus, we utilized recent research findings to cluster individual barriers and strategies more practically and broadly.

### Expert interviews

We aimed to validate the results of our literature review in qualitative expert interviews with GPs, ensuring the relevance and completeness of extracted barriers and improvement strategies to digital health adoption more broadly. Our expert interviews followed the COREQ checklist for qualitative research^65^ (see Supplementary Table 4). We created a semi-structured interview guide based on the findings of our literature review to allow for flexibility yet achieve standardization of the interview procedure (see Supplementary Notes1 for the full interview questionnaire). The questions were designed to capture GPs’ concerns and wishes for digital health adoption as well as their assessment of the relevance of the proposed categories of barriers and strategies. Next to open-ended questions on perceived adoption barriers and relevant improvement strategies, we asked GPs to assess the relevance of the categories of barriers and strategies uncovered in the literature review, i.e., social, organizational, and technological barriers as well as development-related, awareness-related, knowledge-related, implementation-related, and policy-relates strategies. Four topics were covered in detail: (1) experience with digital health solutions, (2) indicators of digital maturity, (3) barriers to digital health adoption, and (4) relevant strategies to improve digital health adoption. This study specifically focuses on the latter two topics, while the first will be part of a separate analysis.

Participants were primarily recruited through targeted sampling and snowballing of personal contacts and colleagues. Participants received information about the research design and topics to be covered in the interview, including a definition of digital health solutions. Participation was voluntary, and written informed consent was obtained from each participant before the interview. The interviews were then conducted virtually in a one-on-one setting, videotaped, and transcribed verbatim to enable further qualitative analysis.

Data saturation was achieved after ten interviews. Participants were 53 years old on average, had been GPs for 18 years, and worked in cities of about 100,000 inhabitants. Four GPs had a solo practice, five worked in a group practice, and one practiced in a medical care center. Interviews lasted 45 min on average.

Coding and qualitative analysis were performed using MAXQDA 2022^67^. To enable a comparison with the findings of our literature review, we developed our coding scheme for our content analysis^68^ deductively based on those findings. To gain further insights, we also inductively inferred themes from the interview material when multiple interviewees brought up the same topic. Based on that, we determined the number of interviewees that indicated the specific barriers or improvement strategy. These results were then compared to the findings of our literature review to develop items for our subsequent online survey. In the survey, we only included items for adoption barriers or improvement strategies proposed by more than four articles or mentioned by more than one interviewee to ensure theoretical and expert consensus.

### Online survey

We next conducted a cross-sectional survey investigating perceived adoption barriers, relevant improvement strategies, and inherent characteristics of GPs. The survey adhered to the CHERRIES checklist for internet surveys^66^ (see Supplementary Table 5 for the completed CHERRIES; see Supplementary Notes2 for a translated version of the survey questionnaire). The survey was divided into six sections: (1) demographics, practice-related characteristics, and digital health usage, (2) GPs’ affinity for technology interaction, (3) Big Five personality traits, (4) digital maturity of the practice, (5) perceived adoption barriers, and (6) relevant improvement strategies. This paper focuses on the findings of sections 1, 2, 3, 5, and 6, as we aimed to investigate the influence of GPs’ inherent characteristics on barriers and improvement strategies. The findings concerning digital maturity were covered in detail in another analysis^69^. Participants were informed of the research objectives, target population, length, and IRB approval on an introductory page. Information regarding data storage and security and the researchers involved were provided on the following page. Before continuing with the survey, participants had to provide informed consent. Afterwards, participants were given a definition of relevant concepts covered in the survey, i.e., digital maturity and digital health solutions.

We captured participants’ demographics and practice-related characteristics using single-choice questions. The current and expected future usage of digital health solutions, as well as the perceived digital affinity of medical assistants were assessed using 5-point Likert-type scales.

To measure GPs’ affinity for technology interaction, we used an established 9-item 6-point Likert-type scale^45^ that captures a person’s tendency to actively participate in intense technology interaction. Using a 21-item German-language measure^46^, we evaluated GPs’ personality traits. The 5-point Likert-type scale evaluates the Big Five personality traits of extraversion, agreeableness, conscientiousness, neuroticism, and openness. Digital maturity was assessed using 28 items with a 5-point Likert-type scale developed in line with a recent systematic review^57^.

We developed the 26 items to assess adoption barriers based on the synthesis of our literature review and expert interview results. Participants were asked to rate their agreement with the items across technological, social, and organizational barriers on a 5-point Likert-type scale. Similarly, the 23 items for our assessment of improvement strategies were also developed based on previous results and captured using a 5-point Likert-type scale. Improvement strategies assessed covered development-related, awareness-related, knowledge-related, implementation-related, and policy-related strategies.

We pretested the survey questionnaire with 15 physicians working in ambulatory care settings to ensure clarity, comprehensiveness, usability, and technical functionality. Question wording and the introductory page were refined after the pre-test. The survey was then conducted between April and mid-August 2023 and took about 10 to 15 min to complete. Various recruitment channels were used to reach a broad sample of German GPs. These included interview participants, personal contacts, teaching practices, physician networks, research practice networks, and GP mailing lists. Participants were contacted via mail using publicly available mail addresses. As we conducted the survey in an open-access mode, anyone with an access link could participate, and we could not track which invited participants had started or completed the survey. We further did not provide incentives for participation.

**Fig. 7:**
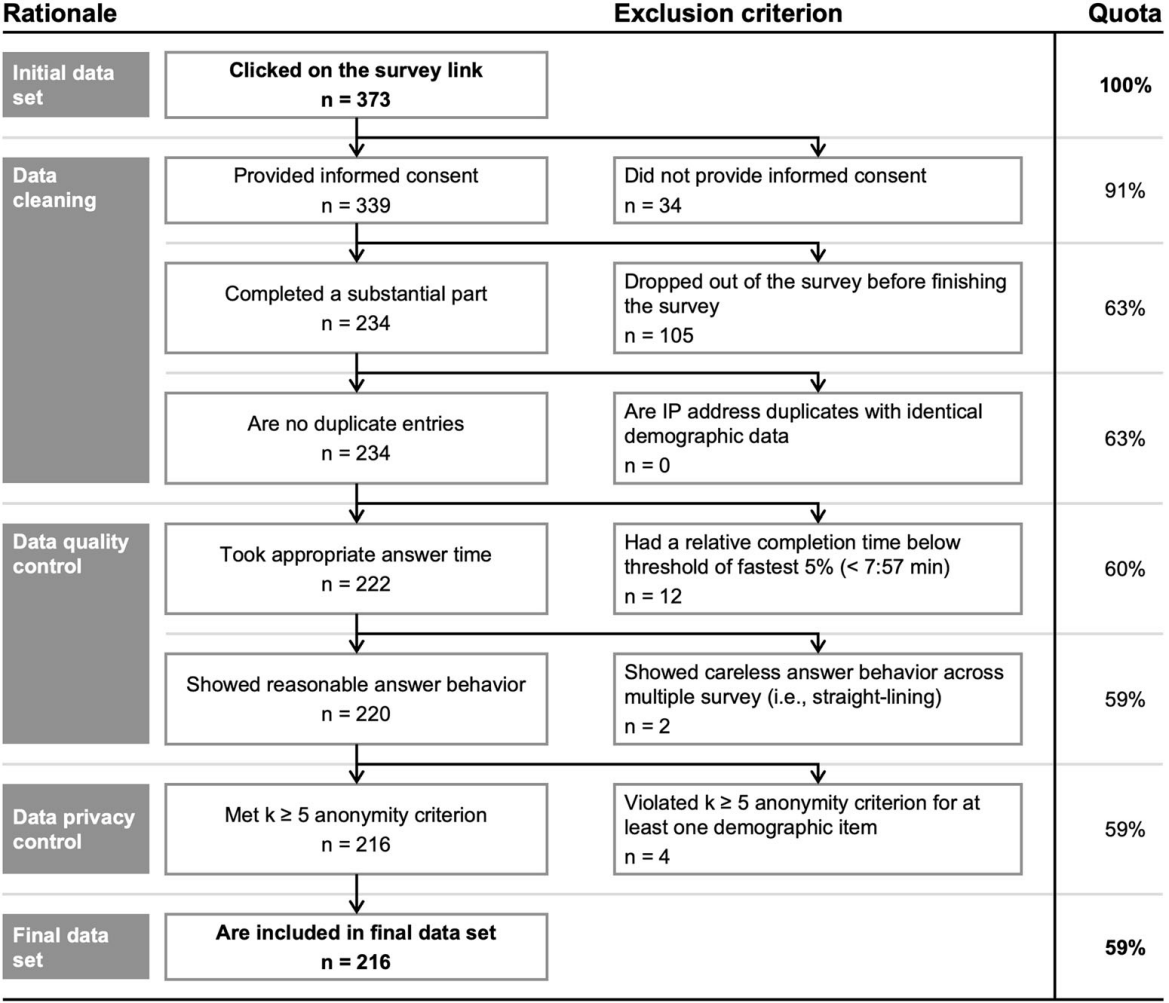
Overview of the data cleaning approach. The figure shows the sequential data cleaning approach, including the number of questionnaires excluded during each process step. As part of our data quality control procedures, we excluded respondents that showed straight-lining across more than two survey pages and thus in more than one item battery, i.e., that chose the very same answer option for all items in more than one item battery, as this might indicate careless responding as opposed to straight-lining due to respondents‘ actual views.

We thoroughly cleaned the data obtained before performing statistical analyses (see Fig. 7). Following standard practice^70^, our data cleaning included removing responses without informed consent, incomplete responses, and duplicate responses. In the next step, we also removed responses that took very little time to complete^71^ and those that displayed careless answer behavior over several survey pages^71^. We further eliminated any responses that did not adhere to our anonymity criterion to comply with data privacy. In total, 216 responses from the 373 people who initially clicked on the survey link are included in our analysis.

For our statistical analyses, we first computed the mean value for respondents’ affinity for technology interaction, their personality traits, their digital maturity, the three categories of adoption barriers, and the five categories of improvement strategies. To allow for an analysis of the influence of GPs’ inherent characteristics on barriers and improvement strategies, we further computed two overall outcome measures for both variables: One outcome measure represents the number of barriers (strategies) and was calculated as the sum of barriers (strategies) that received a score of 4 or higher on our 5-point Likert-type scale and were thus perceived as such. The second outcome measure was calculated as the average across barriers (strategies) and represents the perceived strength of barriers (the perceived importance of strategies). SPSS version 29.0 for Macintosh^72^ was used for all statistical analyses.

We assessed the internal consistencies of the scale used using Cronbach’s Alpha^73^ (see Table 3). Most internal consistencies can be considered as acceptable or good and are in line with previous research^45,46^. However, the internal consistency for conscientiousness was lower in our sample compared to the original study^46^, which might be due to the overall high conscientiousness and low variability of the score in our sample.

**Table 3:** Sample size, mean, standard deviation, number of items, and internal consistencies of the scales used

Scale	Dimension	*n*	mean	SD	# items	α (original)	α (current)
Affinity for technology interaction (ATI)	Affinity for technology interaction	216	3.66	1.08	9	0.89	0.92
Big five personality (BFI-K)	Extraversion	216	3.64	0.80	4	0.86	0.83
Big five personality (BFI-K)	Agreeableness	216	3.53	0.75	4	0.64	0.68
Big five personality (BFI-K)	Conscientiousness	216	4.10	0.59	4	0.70	0.61
Big five personality (BFI-K)	Neuroticism	216	2.42	0.71	4	0.74	0.72
Big five personality (BFI-K)	Openness	216	3.85	0.68	5	0.66	0.73
Perceived barriers	Technological	213	2.93	0.76	7	-	0.76
Perceived barriers	Social	215	2.76	0.79	8	-	0.84
Perceived barriers	Organizational	215	3.56	0.71	11	-	0.84
Improvement strategies	Development-related	215	3.98	0.67	6	-	0.79
Improvement strategies	Awareness-related	216	3.70	0.74	4	-	0.72
Improvement strategies	Knowledge-related	216	3.85	0.81	4	-	0.84
Improvement strategies	Implementation-related	216	3.90	0.78	5	-	0.77
Improvement strategies	Policy-related	216	4.00	0.81	4	-	0.74

The table provides an overview of sample sizes, means, standard deviations, items, and internal consistencies of the scales used in the study. All items and subscales to assess perceived barriers and improvement strategies were developed based on the literature review and expert interview results. Thus, Cronbach’s Alpha for the original studies cannot be shown.

Given the several inherent variables pertinent to our study, we conducted independent univariate ANOVAs with 2-tailed significance (*p* < 0.05; Welch ANOVA) to compare differences in barriers and strategies. Welch’s F^74^ was used as a robust measure for all ANOVAs because some of our variables did not follow a normal distribution, as demonstrated by Q-Q-Plots, Shapiro–Wilk’s test, and were heteroscedastic in some cases as indicated by Levene’s test. Were ANOVAs showed a significant omnibus difference (*p* < 0.05; Welch ANOVA), we looked at Hochberg’s GT2 (homogeneity of variance met) or Games-Howell (homogeneity of variance not met) as post hoc procedures^75^. In addition, we utilized Cohen’s d as a measure of effect size^76^. As we aimed to assess differences in barriers and strategies based on digital affinity and personality, we grouped participants into three categories (low, moderate, high) for each characteristic based on theoretical thirds of the underlying Likert-type scales. As this categorization does not cover the whole spectrum of the continuous underlying variable, it was only used in our ANOVAs as an initial indicator for differences in these variables between GPs. These differences were then analyzed more granularly in our regression model utilizing the continuous variables without categorization.

We conducted a linear hierarchical regression analysis to deepen our understanding of the association between barriers and strategies and the independent variables, further accounting for the continuity associated with personality and digital affinity-related variables. We chose a hierarchical approach for entering variables into our model to determine the influence of demographic and practice-related variables on barriers and strategies and to separate this from the influence of digital health usage, digital affinity, and personality. Potential multicollinearity of predictors was assessed following practical recommendations using VIF and tolerance values^75^. As all VIF values were below ten and tolerance values greater than 0.1, multicollinearity does not seem to flaw our analysis. In our sequential approach, the first stage incorporated demographics and practice-related characteristics, including gender, age, practice location size, professional experience, and practice type. The second stage introduced variables related to digital health usage – current and expected future usage. The third stage added digital affinity-related variables, encompassing the perceived digital affinity of medical assistants and GPs affinity for technology interaction. The fourth stage introduced the Big Five personality traits of extraversion, agreeableness, conscientiousness, neuroticism, and openness. In the final model, the digital maturity of the practice was included. This sequence followed prior research and theoretical reasoning, with variables analyzed in past research entering earlier in the model. In addition, the distinct blocks analyzed covered different categories of inherent variables, namely demographics, practice-related characteristics, digital health usage, digital affinity, and personality. As our analysis of the relationship between perceived barriers and strategies and inherent characteristics was novice, our analysis focused on main effects of individual predictor variables and explicitly refrained from analyzing and interpreting interaction effects.

### Supplementary information

Supplementary Material

## Data Availability

The data supporting this study’s findings are available from the corresponding author upon reasonable request.
