# Supplementary material for: Understanding inherent influencing factors to digital health adoption in general practices through a mixed-methods analysis
Source: NPJ Digit Med. 2024 Feb 27;7:47. doi: 10.1038/s41746-024-01049-0 (PMC10899241; doi:10.1038/s41746-024-01049-0)
Supplement: Supplementary file 1 — Supplementary Material [file 41746_2024_1049_MOESM1_ESM.pdf]

Supplementary Table 1. Overview of regression coefficients for the final linear hierarchical regression model predicting the strength of adoption barriers (model 5).

Variable	b	SE B	β	P value	95% CI
**(Constant)**	5.214	0.510	-	<.001	4.21 , 6.22
**Gender: female vs. male**	0.011	0.089	.008	.905	-0.16 , 0.19
**Age: 36-45 vs. 26-35**	0.087	0.162	.057	.589	-0.23 , 0.41
**Age: 46-55 vs. 26-35**	0.055	0.195	.036	.777	-0.33 , 0.44
**Age: 56-65 vs. 26-35**	0.031	0.236	.022	.896	-0.44 , 0.50
**Age: >65 vs. 26-35**	0.106	0.320	.031	.742	-0.53 , 0.74
**Location: 5,000-20,000 vs. <5,000**	-0.129	0.117	-.090	.272	-0.36 , 0.10
**Location: 20,001-100,000 vs. <5,000**	-0.227	0.129	-.132	.080	-0.48 , 0.03
**Location: 100,001-500,000 vs. <5,000**	-0.315	0.142	-.152	.028	-0.60 , -0.03
**Location: >500,000 vs. <5,000**	-0.301	0,133	-.164	.025	-0.56 , -0.04
**Experience: 6-10 vs. 1-5**	0.004	0.157	.002	.978	-0.31 , 0.31
**Experience: 11-20 vs. 1-5**	-0.008	0.160	-.005	.959	-0.32 , 0.31
**Experience: 21-30 vs. 1-5**	0.257	0.200	.170	.200	-0.14 , 0.65
**Experience: >30 vs. 1-5**	0.168	0.236	.101	.478	-0.30 , 0.63
**Type: Practice sharing vs. single practice**	0.498	0.189	.155	.009	0.13 , 0.87
**Type: Group practice vs. single practice**	-0.114	0.086	-.084	.186	-0.28 , 0.06
**Type: Medical care center vs. single practice**	-0.100	0.189	-.033	.596	-0.47 , 0.27
**Current usage: less than once per month vs. never**	-0.081	0.158	-.049	.609	-0.39 , 0.23
**Current usage: monthly vs. never**	-0.020	0.201	-.009	.923	-0.42 , 0.38
**Current usage: weekly vs. never**	-0.144	0.194	-.076	.461	-0.53 , 0.24
**Current usage: daily vs. never**	0.074	0.198	.055	.707	-0.32 , 0.46
**Future usage**	-0.151	0.051	-.281	.003	-0.25 , -0.05
**Perceived digital affinity of medical assistants**	-0.025	0.044	-.037	.564	-0.11 , 0.06
**General practitioners' affinity for technology interaction**	-0.159	0.042	-.254	<.001	-0.24 , -0.08
**Extraversion**	-0.109	0.055	-.129	.048	-0.22 , 0.00
**Agreeableness**	-0.084	0.054	-.093	.123	-0.19 , 0.02
**Conscientiousness**	-0.046	0.075	-.040	.535	-0.19 , 0.10
**Neuroticism**	0.156	0.063	.164	.014	0.03 , 0.28
**Openness**	0.134	0.062	.135	.031	0.01 , 0.26
**Digital maturity**	-0.247	0.071	-.236	.001	-0.39 , -0.11

Note: SE = standard error for coefficient b; 95% CI = upper and lower bound for the 95% confidence interval.

Supplementary Table 2. Overview of regression coefficients for the linear hierarchical regression model predicting the importance of improvement strategies (model 2).

Variable	b	SE B	β	*P* value	95% CI
**(Constant)**	3.086	0.284	-	<.001	2.53 , 3.65
**Gender: female vs. male**	0.129	0.087	.104	.141	-0.04 , 0.30
**Age: 36-45 vs. 26-35**	0.179	0.166	.128	.280	-0.15 , 0.51
**Age: 46-55 vs. 26-35**	0.275	0.200	.196	.170	-0.12 , 0.67
**Age: 56-65 vs. 26-35**	0.188	0.242	.146	.438	-0.29 , 0.67
**Age: >65 vs. 26-35**	0.631	0.331	.206	.058	-0.02 , 1.28
**Location: 5,000-20,000 vs. <5,000**	0.065	0.122	.050	.594	-0.17 , 0.30
**Location: 20,001-100,000 vs. <5,000**	0.090	0.133	.058	.500	-0.17 , 0.35
**Location: 100,001-500,000 vs. <5,000**	-0.089	0.150	-.047	.551	-0.38 , 0.21
**Location: >500,000 vs. <5,000**	-0.107	0.135	-.064	.431	-0.37 , 0.16
**Experience: 6-10 vs. 1-5**	-0.145	0.165	-.083	.381	-0.47 , 0.18
**Experience: 11-20 vs. 1-5**	-0.231	0.167	-.167	.169	-0.56 , 0.10
**Experience: 21-30 vs. 1-5**	-0.524	0.210	-.381	.013	-0.94 , -0.11
**Experience: >30 vs. 1-5**	-0.477	0.246	-.316	.054	-0.96 , 0.01
**Type: practice sharing vs. single practice**	-0.011	0.200	-.004	.956	-0.40 , 0.38
**Type: group practice vs. single practice**	-0.091	0.091	-.073	.318	-0.27 , 0.09
**Type: medical care center vs. single practice**	-0.014	0.198	-.005	.945	-0.40 , 0.38
**Current usage: less than once per month vs. never**	0.458	0.165	.308	.006	0.13 , 0.78
**Current usage: monthly vs. never**	0.430	0.208	.221	.040	0.02 , 0.84
**Current usage: weekly vs. never**	0.247	0.204	.143	.227	-0.15 , 0.65
**Current usage: daily vs. never**	0.326	0.207	.264	.116	-0.08 , 0.73
**Future usage**	0.105	0.052	.216	.043	0.00 , 0.21

Note: SE = standard error for coefficient b; 95% CI = upper and lower bound for the 95% confidence interval.

Supplementary Table 3. PRISMA extension for scoping reviews (PRISMA-ScR) checklist.

SECTION	ITEM	PRISMA-ScR CHECKLIST ITEM	REPORTED IN SECTION
**TITLE**			
Title	1	Identify the report as a scoping review.	n/a
**ABSTRACT**			
Structured summary	2	Provide a structured summary that includes (as applicable): background, objectives, eligibility criteria, sources of evidence, charting methods, results, and conclusions that relate to the review questions and objectives.	Background: Extensive research has already shown the potential value of digital health solutions and highlights the importance of clinicians' adoption. As general practitioners (GPs) are patients' first point of contact, identifying barriers to adoption and potential strategies for improvement is especially important. In this context, extensive research has studied digital health adoption across various medical disciplines, healthcare settings, and technologies. However, only a few studies considered a broader perspective on adopting digital health solutions and investigated potential strategies to improve adoption. Thus, we aimed to identify and synthesize relevant barriers and improvement strategies across technologies relevant to general practice settings.<br><br>Methods: For our scoping review, we searched the PubMed and PsycINFO databases on 20 February 2023 and manually de-duplicated the results. We narrowed initially identified citations to studies published in English or German between January 2018 and December 2022 to account for more recent research findings. We included all articles that focused on clinician populations, digital health solutions, and general practice settings that addressed, measured, and reported factors impacting or promoting the adoption or use of digital health solutions. We included only peer-reviewed articles, as we aimed to identify validated barriers and strategies. We synthesized evidence from the included studies by extracting and grouping potentially relevant barriers and improvement strategies to digital health adoption.<br><br>Findings: Of 1276 records initially identified in the two databases, 24 were eligible. Most studies were quantitative (11/24; 46%) and focused on GPs' (17/24; 71%) adoption of mHealth applications

SECTION	ITEM	PRISMA-ScR CHECKLIST ITEM	REPORTED IN SECTION
			(6/24; 25%). While there was a large variability of digital health solutions targeted in the studies, most did not look at specific conditions to be treated (16/24; 67%) or utilized theoretical models to describe digital health adoption (16/24; 67%). More than 90% of included studies report organizational barriers to digital health adoption (23/24), with more than half demonstrating that high workload and a lack of time (each 14/23; 61%) are predominant barriers to adoption. Another 88% of studies identify social adoption barriers (21/24). Of these, GPs’ familiarity with digital health solutions (17/21; 81%) was the most cited barrier to adoption, followed by overall awareness (10/21; 48%) and patient preferences (10/21; 48%). Looking into potential strategies to support digital health adoption, around two-thirds of studies identify strategies related to developing digital health solutions as potentially helpful to improve adoption (16/24; 67%). Among these, the most frequently cited aspects are those that improve the usefulness of digital health solutions (13/21; 62%)<br><br>Interpretation: Many studies investigate the adoption barriers of GPs regarding digital health solutions. However, they primarily focus on dedicated technologies and adoption barriers instead of an integrated perspective. To date, many adoption barriers have been identified, while only a few studies investigate potential strategies to support the adoption by GPs.
**INTRODUCTION**			
Rationale	3	Describe the rationale for the review in the context of what is already known. Explain why the review questions/objectives lend themselves to a scoping review approach.	Extensive research has already shown the potential value of digital health and the importance of GPs' adoption regarding the success of these technologies. Since GPs are patients' first point of contact, identifying factors impacting and promoting their adoption of digital health is crucial. Many studies have identified barriers and improvement strategies for dedicated digital solutions. However, most of these have applied an overarching perspective, while these barriers might be impacted by factors inherent to the GP. Thus, this review aims to provide a comprehensive understanding of barriers and improvement strategies to allow a subsequent mixed-methods study to investigate the impact of GPs' inherent characteristics.

SECTION	ITEM	PRISMA-ScR CHECKLIST ITEM	REPORTED IN SECTION
Objectives	4	Provide an explicit statement of the questions and objectives being addressed with reference to their key elements (e.g., population or participants, concepts, and context) or other relevant key elements used to conceptualize the review questions and/or objectives.	Our review aimed to more broadly identify currently postulated perceived barriers and potential improvement strategies for digital health adoption across technologies. With this, a subsequent mixed-methods study would allow us to (1) create transparency on perceived barriers and improvement strategies to digital health adoption of GPs in Germany and (2) assess the role of personality traits and other inherent characteristics as underlying influencing factors.
**METHODS**			
Protocol and registration	5	Indicate whether a review protocol exists; state if and where it can be accessed (e.g., a Web address); and if available, provide registration information, including the registration number.	As this review was part of a mixed-methods approach, we did not prepare, register, and publish a review protocol.
Eligibility criteria	6	Specify characteristics of the sources of evidence used as eligibility criteria (e.g., years considered, language, and publication status), and provide a rationale.	see 'Literature Review'
Information sources	7	Describe all information sources in the search (e.g., databases with dates of coverage and contact with authors to identify additional sources), as well as the date the most recent search was executed.	see 'Literature Review'
Search	8	Present the full electronic search strategy for at least 1 database, including any limits used, such that it could be repeated.	To identify potentially relevant articles for our research in both databases, we developed a search string covering four categories of keywords: (1) adoption (adoption OR implementation OR utilization OR utilisation OR experience* OR usage OR use OR uptake OR spread OR introduction); (2) digital health (mHealth OR "m Health" OR m-health OR "mobile health" OR eHealth OR "e Health" OR e-health OR telehealth OR "health information system*" OR "digital health" OR "mobile app*" OR smartphone* OR telemonitoring OR "remote monitoring" OR "virtual care" OR technolog* OR internet* OR "mobile device"); (3) barriers/facilitators (barrier* OR hinder* OR prevent* OR limit* OR facilitat* OR improve* OR support* OR promot* OR strateg* OR incentiv*). For a more targeted view of digital health adoption in general practices, we added the following MeSH Terms to our search string: (general practitioners) OR (physicians, family) OR

SECTION	ITEM	PRISMA-ScR CHECKLIST ITEM	REPORTED IN SECTION
			(physicians, primary care) OR (physicians, women) OR (general practice) OR (family practice). We applied the search string to the Title/Abstract field in PubMed and full-text fields for PsycINFO. We narrowed initially identified citations to studies published in English or German between January 2018 and December 2022 to account for more recent research findings.
Selection of sources of evidence	9	State the process for selecting sources of evidence (i.e., screening and eligibility) included in the scoping review.	see 'Literature Review'
Data charting process	10	Describe the methods of charting data from the included sources of evidence (e.g., calibrated forms or forms that have been tested by the team before their use, and whether data charting was done independently or in duplicate) and any processes for obtaining and confirming data from investigators.	We developed a data extraction sheet listing all the relevant data we needed to extract from the eligible studies. According to the sheet, LW extracted all data from eligible studies. All data were extracted as reported in the studies and compared after extraction to harmonize wording across studies.
Data items	11	List and define all variables for which data were sought and any assumptions and simplifications made.	We sought data for all factors impacting or promoting digital health adoption reported in the eligible studies, i.e., that were reported as part of a quantitative assessment or a theoretical model. In addition, we sought data on study-related characteristics. We extracted information from each eligible report on (1) the report itself (including author, publication year, journal, and publication identifier); (2) the study itself (including sample characteristics, sample size, and study location); (3) the research design (including the study design and theoretical framework utilized); (4) the intervention (i.e., the type of digital health solution under consideration and the condition treated).
Critical appraisal of individual sources of evidence	12	If done, provide a rationale for conducting a critical appraisal of included sources of evidence; describe the methods used and how this information was used in any data synthesis (if appropriate).	We did not conduct a dedicated critical appraisal of included studies as we aimed to identify factors impacting or promoting digital health adoption as presented in multiple studies and only included evidence from more than one study and aimed to validate the results of our literature review in qualitative expert interviews with GPs, ensuring relevance and completeness of extracted barriers and improvement strategies.
Synthesis of results	13	Describe the methods of handling and summarizing the data that were charted.	see 'Literature Review'
**RESULTS**			

SECTION	ITEM	PRISMA-ScR CHECKLIST ITEM	REPORTED IN SECTION
Selection of sources of evidence	14	Give numbers of sources of evidence screened, assessed for eligibility, and included in the review, with reasons for exclusions at each stage, ideally using a flow diagram.	see ‘Literature Review’ and ‘Figure 6’
Characteristics of sources of evidence	15	For each source of evidence, present characteristics for which data were charted and provide the citations.	see ‘Adoption barriers and improvement strategies in general practices (literature review and expert interview results)’<br><br>The detailed characteristics of included studies and the extracted data mentioned above can be obtained from the corresponding author upon request and is not presented in detail.
Critical appraisal within sources of evidence	16	If done, present data on critical appraisal of included sources of evidence (see item 12).	n/a
Results of individual sources of evidence	17	For each included source of evidence, present the relevant data that were charted that relate to the review questions and objectives.	‘Adoption barriers and improvement strategies in general practices (literature review and expert interview results)’
Synthesis of results	18	Summarize and/or present the charting results as they relate to the review questions and objectives.	‘Adoption barriers and improvement strategies in general practices (literature review and expert interview results)’ and ‘Figure 1’
**DISCUSSION**			
Summary of evidence	19	Summarize the main results (including an overview of concepts, themes, and types of evidence available), link to the review questions and objectives, and consider the relevance to key groups.	see ‘Adoption barriers and improvement strategies in general practices (literature review and expert interview results)’
Limitations	20	Discuss the limitations of the scoping review process.	First, as we limited our search to peer-reviewed articles published in German or English and excluded grey literature, we might not have identified all literature relevant to our research question and potentially encountered a publication bias. In addition, our inclusion and exclusion criteria defined before our search also limited our approach.<br>Second, as we further limited our search to articles published in the last five years, we might have missed older evidence. Thus, we might not have captured all factors impacting or promoting digital health adoption proposed in the literature. As the COVID-19 pandemic has accelerated the adoption of digital health, we aimed at capturing more recent evolvements.

SECTION	ITEM	PRISMA-ScR CHECKLIST ITEM	REPORTED IN SECTION
			Third, as our review was part of a more extensive mixed-methods study, our search, data extraction, and synthesis were solely conducted by one author and not validated by a second reviewer. We are confident, that this would not have changed our synthesis of results as we validated these externally via expert interviews.
Conclusions	21	Provide a general interpretation of the results with respect to the review questions and objectives, as well as potential implications and/or next steps.	Many studies investigate GPs' adoption barriers regarding digital health solutions. However, they mainly focus on dedicated technologies and conditions instead of an integrated perspective. This finding aligns with other reviews targeting digital health adoption for dedicated technologies. Many adoption barriers have been identified, while only a few studies look into potential strategies to support adoption. However, recent reviews highlight the importance of social factors for digital health adoption, which we echo in our findings. As no single approach to enhancing digital health adoption applies to all GPs, we call for analyzing personality and other inherent characteristics as potential influencing factors on digital health adoption. We believe that exploring personality as a potential underlying factor relevant to digital health adoption is particularly important in general practices as (1) digitalization in general practices is mainly driven by GPs themselves and thus will not be pursued unless they deem it necessary, (2) there is already some evidence highlighting the role of personality for patients' adoption of digital health solutions.
**FUNDING**			
Funding	22	Describe sources of funding for the included sources of evidence, as well as sources of funding for the scoping review. Describe the role of the funders of the scoping review.	As this research is part of a doctoral thesis at Witten/Herdecke University, it has not received any funding.

Supplementary Table 4. Consolidated criteria for reporting qualitative research (COREQ) checklist.

DOMAIN	ITEM	GUIDE QUESTIONS / DESCRIPTIONS	REPORTED IN SECTION
**DOMAIN 1: RESEARCH TEAM AND REFLEXIVITY**			
Personal characteristics			
Interviewer/facilitator	1	Which author/s conducted the interview or focus group?	see 'Author contributions'
Credentials	2	What were the researcher's credentials? (e.g., PhD, MD)	LW holds a Master of Science in Psychology.
Occupation	3	What was their occupation at the time of the study?	LW is a Ph.D. candidate.
Gender	4	Was the researcher male or female?	LW is female.
Experience and training	5	What experience or training did the researcher have?	LW was experienced in conducting interviews based on her educational background in psychology and her professional background in strategy consulting.
Relationship with participants			
Relationship established	6	Was a relationship established prior to study commencement?	There was no relationship established to the participants prior to the study.
Participant knowledge of the interviewer	7	What did the participants know about the researcher? (e.g., personal goals, reasons for doing the research)	In the beginning of the interview, participants were informed about the educational and professional background of the researcher, and the overall objectives and aims of the research.
Interviewer characteristics	8	What characteristics were reported about the interviewer/facilitator? (e.g., bias, assumptions, reasons, and interests in the research topic)	Participants were informed that the interest in the research topic was based on the researcher's professional background.
**DOMAIN 2: STUDY DESIGN**			
Theoretical framework			
Methodological orientation and theory	9	What methodological orientation was stated to underpin the study? (e.g., grounded theory, discourse analysis, ethnography, phenomenology, content analysis)	see 'Study design' and 'Expert interviews'
Participant selection			
Sampling	10	How were participants selected? (e.g., purposive, convenience, consecutive, snowball)	see 'Expert interviews'
Method of approach	11	How were participants approached? (e.g., face-to-face, telephone, mail, email)	see 'Expert interviews'

DOMAIN	ITEM	GUIDE QUESTIONS / DESCRIPTIONS	REPORTED IN SECTION
Sample size	12	How many participants were in the study?	see 'Expert interviews'
Non-participation	13	How many people refused to participate or dropped out? Reasons?	Initially we approached 26 general practitioners, of which 15 did not respond to our interview enquiry. One additional general practitioner declined to be interviewed due to a lack of time and high workload.
Setting			
Setting of data collection	14	Where was the data collected? (e.g., home, clinic, workplace)	see 'Expert interviews'
Presence of non-participants	15	Was anyone else present besides the participants and researchers?	see 'Expert interviews'
Description of sample	16	What are important characteristics of the sample? (e.g., demographic data, date)	see 'Expert interviews'<br><br>As we purposively sampled participants, we were able to include diverse age ranges and work experience levels as well as locations and practice types. However only 20% of interviewees were female. On average, participants were 53 years old and have worked as a general practitioner for 18 years in a city with 105k inhabitants. Four general practitioners worked in a single practice, five worked in a group practice and one worked in a medical care center.
Data collection			
Interview guide	17	Were questions, prompts, guides provided by the authors? Was it pilot tested?	see 'Expert interviews'<br><br>We utilized a semi-structured interview guide for the interviews to allow for flexibility yet achieve standardization of the interview procedure.
Repeat interviews	18	Were repeat interviews carried out? If yes, how many?	No repeat interviews were carried out.
Audio/visual recording	19	Did the research use audio or visual recording to collect the data?	see 'Expert interviews'
Field notes	20	Were field notes made during and/or after the interview or focus group?	No field notes were made during the interviews. However, a short summary of the interview was written after transcription.
Duration	21	What was the duration of the interviews or focus group?	see 'Expert interviews'
Data saturation	22	Was data saturation discussed?	see 'Expert interviews'

DOMAIN	ITEM	GUIDE QUESTIONS / DESCRIPTIONS	REPORTED IN SECTION
			We planned to interview between 8 and 15 general practitioners based on data saturation. This was achieved, i.e., no new content on top of the derived themes based on the literature research emerged by the time we interviewed 10 general practitioners.
Transcripts returned	23	Were transcripts returned to participants for comment and/or correction?	We did not return transcripts to participants.
**DOMAIN 3: ANALYSIS AND FINDINGS**			
Data analysis			
Number of data coders	24	How many data coders coded the data?	see 'Author contributions'
Description of the coding tree	25	Did authors provide a description of the coding tree?	see 'Expert interviews'
Derivation of themes	26	Were themes identified in advance or derived from the data?	see 'Expert interviews'
Software	27	What software, if applicable, was used to manage the data?	see 'Expert interviews'
Participant checking	28	Did participants provide feedback on the findings?	We did not ask for feedback on the findings from participants.
Reporting			
Quotations presented	29	Were participant quotations presented to illustrate the themes/findings? Was each quotation identified? (e.g., participant number)	We do not present participant quotes.
Data and findings consistent	30	Was there consistency between the data presented and the findings?	Data presented and findings are consistent.
Clarity of major themes	31	Were major themes clearly presented in the findings?	see 'Adoption barriers and improvement strategies in general practices (literature review and expert interview results)'<br><br>The section provides a quantitative overview of interview themes and their contrast with literature review results. We do not discriminate between major or minor themes.
Clarity of minor themes	32	Is there a description of diverse cases or discussion of minor themes?	see 'Adoption barriers and improvement strategies in general practices (literature review and expert interview results)'

Supplementary Table 5. Checklist for reporting results of internet e-surveys (CHERRIES).

ITEM CATEGORY	ITEM	EXPLANATION	REPORTED IN SECTION
**DESIGN**			
Describe survey design	1	Describe target population, sample frame. Is the sample a convenience sample? (In “open” surveys this is most likely.)	see ‘Online survey’
**IRB (Institutional Review Board) APPROVAL AND INFORMED CONSENT PROCESS**			
IRB approval	2	Mention whether the study has been approved by an IRB.	see ‘Online survey’
Informed consent	3	Describe the informed consent process. Where were the participants told the length of time of the survey, which data were stored and where and for how long, who the investigator was, and the purpose of the study?	see ‘Online survey’
Data protection	4	If any personal information was collected or stored, describe what mechanisms were used to protect unauthorized access.	Data were anonymized following the principle of k-anonymity and stored solely on the Witten/Herdecke University server. We did not collect any personal information that could be tied back to individual respondents.
**DEVELOPMENT AND PRE-TESTING**			
Development and testing	5	State how the survey was developed, including whether the usability and technical functionality of the electronic questionnaire had been tested before fielding the questionnaire.	see ‘Online survey’
**RECRUITMENT PROCESS AND DESCRIPTION OF THE SAMPLE HAVING ACCESS TO THE QUESTIONNAIRE**			
Open survey versus closed survey	6	An “open survey” is a survey open for each visitor of a site, while a closed survey is only open to a sample which the investigator knows (password-protected survey).	see ‘Online survey’
Contact mode	7	Indicate whether or not the initial contact with the potential participants was made on the Internet. (Investigators may also send out questionnaires by mail and allow for Web-based data entry.)	see ‘Online survey’
Advertising the survey	8	How/where was the survey announced or advertised? Some examples are offline media	see ‘Online survey’

ITEM CATEGORY	ITEM	EXPLANATION	REPORTED IN SECTION
		(newspapers), or online (mailing lists – If yes, which ones?) or banner ads (Where were these banner ads posted and what did they look like?). It is important to know the wording of the announcement as it will heavily influence who chooses to participate. Ideally the survey announcement should be published as an appendix.	The announcements used for recruitment varied slightly in length and style to match the audience and channel. Overall, we provided the different channels with a formal digital letter signed by the research team as an official invitation to participate in the survey, a digital flyer containing the most relevant information about the survey (i.e., research question, target group, survey duration), and the respective survey link. The formal letter included the following message (translated English version): "Dear Sir or Madam, The Chair of Health Informatics at Witten/Herdecke University is currently looking for participants for an online survey entitled 'digitalization in general practice settings - influencing factors and perceived barriers'. In the research project, the team investigates the degree of digitalization in German general practices, perceived barriers, and potential strategies for improvement concerning the adoption of digital health solutions. The survey, which takes about 15 minutes to complete, is aimed at practicing general practitioners with a practice location in Germany, regardless of their experience with digital health solutions. You are welcome to follow this link to participate in the survey: [survey link]. If you have any questions or suggestions, please feel free to contact the chair team [mail address].".<br><br>On LinkedIn, the survey was shared with the following message (translated English version): "Digitalization in general practices and the human factor - obstacle or success factor? The Chair of Health Informatics at Witten/Herdecke University currently investigates this question in a short online survey. Our research aims to assess the degree of digitalization of German general practices, record perceived barriers to adopting digital health solutions, and further investigate the relationship with personal characteristics. The 15-minute survey targets practicing general practitioners with practices in Germany, regardless of their experience with digital health solutions. You are welcome to participate at: [survey link].".
**SURVEY ADMINISTRATION**			

ITEM CATEGORY	ITEM	EXPLANATION	REPORTED IN SECTION
Web/E-Mail	9	State the type of e-survey (e.g., one posted on a Web site, or one sent out through e-mail). If it is an e-mail survey, were the responses entered manually into a database, or was there an automatic method for capturing responses?	The survey was administered solely through the web. We utilized the online survey tool LimeSurvey to create and launch the survey.
Context	10	Describe the Web site (for mailing list/newsgroup) in which the survey was posted. What is the Web site about, who is visiting it, what are visitors normally looking for? Discuss to what degree the content of the Web site could pre-select the sample or influence the results. For example, a survey about vaccination on an anti-immunization Web site will have different results from a Web survey conducted on a government Web site	n/a
Mandatory/voluntary	11	Was it a mandatory survey to be filled in by every visitor who wanted to enter the Web site, or was it a voluntary survey?	see ‘Online survey’
Incentives	12	Were any incentives offered (e.g., monetary, prizes, or non-monetary incentives such as an offer to provide the survey results)?	see ‘Online survey’
Time/date	13	In what timeframe were the data collected?	see ‘Online survey’
Randomization of items or questionnaires	14	To prevent biases items can be randomized or alternated.	We did not randomize items as we chose the sequence of questions based on general questionnaire design guidelines. We asked participants to answer questions on their demographics, practice-related characteristics, digital health usage, affinity for technology interaction, and personality first (i.e., independent variables), before they had to answer the remaining items for dependent variables (i.e., digital maturity, adoption barriers, strategies to support adoption).
Adaptive questioning	15	Use adaptive questioning (certain items, or only conditionally displayed based on responses to other items) to reduce number and complexity of the questions.	As we partially utilized existing instruments, we did not use adaptive questioning in our survey.
Number of items	16	What was the number of questionnaire items per page? The number of items is an important factor for the completion rate.	see ‘Online survey’<br><br>The total number of questions was 17, with demographics, practice-related characteristics, and digital health usage

ITEM CATEGORY	ITEM	EXPLANATION	REPORTED IN SECTION
			containing nine single-choice questions, affinity for technology interaction and personality each containing one Likert-type scale, and the remaining three sections each being spread across two survey pages and thus containing two Likert-type scales each. Across all questions our survey consisted of 116 items.
Number of screens (pages)	17	Over how many pages was the questionnaire distributed? The number of items is an important factor for the completion rate.	In general, sections (1) to (3) were displayed on separate questionnaire pages, while sections (4) to (6) were each split into two pages to reduce the number of items per page and allow for a more user-friendly completion of the questionnaire. In total, the survey was distributed over 12 pages, with nine questionnaire pages covering the different sections, one welcome and goodbye screen, and one screen to provide informed consent.
Completeness check	18	It is technically possible to do consistency or completeness checks before the questionnaire is submitted. Was this done, and if "yes", how (usually JAVAScript)? An alternative is to check for completeness after the questionnaire has been submitted (and highlight mandatory items). If this has been done, it should be reported. All items should provide a non-response option such as "not applicable" or "rather not say", and selection of one response option should be enforced.	We utilized forced responses for the first three sections and soft-forced-response (i.e., reminders to provide complete answers) for the latter. To enforce the selection of one response option and not undermine the forced response of the question, the survey did not provide an "I don't know" option. We applied both strategies to gain complete, meaningful responses, even though this might have led to a lower response rate.
Review step	19	State whether respondents were able to review and change their answers (e.g., through a Back button or a Review step which displays a summary of the responses and asks the respondents if they are correct).	We decided not to include a "back" button for respondents to review and change their responses to counter a potential social desirability effect that might cause them to change their responses after completing subsequent questions.
**RESPONSE RATES**			
Unique site visitors	20	If you provide view rates or participation rates, you need to define how you determined a unique visitor. There are different techniques available, based on IP addresses or cookies or both.	see 'Online survey' and 'Figure 7'
View rate (Ratio of unique survey visitors/unique site visitors)	21	Requires counting unique visitors to the first page of the survey, divided by the number of unique site visitors (not page views!). It is not unusual to have	n/a

ITEM CATEGORY	ITEM	EXPLANATION	REPORTED IN SECTION
		view rates of less than 0.1 % if the survey is voluntary.	
Participation rate (ratio of those who agreed to participate/unique first survey page visitors)	22	Count the unique number of people who filled in the first survey page (or agreed to participate, for example by checking a checkbox), divided by visitors who visit the first page of the survey (or the informed consents page, if present). This can also be called "recruitment" rate.	see 'Online survey' and 'Figure 7'
Completion rate (ratio of users who finished the survey/users who agreed to participate)	23	The number of people submitting the last questionnaire page, divided by the number of people who agreed to participate (or submitted the first survey page). This is only relevant if there is a separate "informed consent" page or if the survey goes over several pages. This is a measure for attrition. Note that "completion" can involve leaving questionnaire items blank. This is not a measure for how completely questionnaires were filled in. (If you need a measure for this, use the word "completeness rate".)	see 'Online survey' and 'Figure 7'
**PREVETING MULTIPLE ENTRIES FROM THE SAME INDIVIDUAL**			
Cookies used	24	Indicate whether cookies were used to assign a unique user identifier to each client computer. If so, mention the page on which the cookie was set and read, and how long the cookie was valid. Were duplicate entries avoided by preventing users access to the survey twice; or were duplicate database entries having the same user ID eliminated before analysis? In the latter case, which entries were kept for analysis (e.g., the first entry or the most recent)?	We decided not to use cookies to prevent multiple entries from the same individual, as general practitioners in one practice might share a computer. Thus, we would automatically exclude them from participating in the survey.
IP check	25	Indicate whether the IP address of the client computer was used to identify potential duplicate entries from the same user. If so, mention the period of time for which no two entries from the same IP address were allowed (e.g., 24 hours). Were duplicate entries avoided by preventing users with	see 'Online survey' and 'Figure 7'<br><br>We checked the anonymized IP addresses of participants to identify duplicate entries from the same individual. However, we only excluded IP address duplicates with identical demographic data to ensure that we would allow general practitioners working

ITEM CATEGORY	ITEM	EXPLANATION	REPORTED IN SECTION
		the same IP address access to the survey twice; or were duplicate database entries having the same IP address within a given period of time eliminated before analysis? If the latter, which entries were kept for analysis (e.g., the first entry or the most recent)?	in the same practice and thus sharing an IP address to participate in the survey. We kept the most recent and complete entry for the cases with identical IP addresses and demographic data.
Log file analysis	26	Indicate whether other techniques to analyze the log file for identification of multiple entries were used. If so, please describe.	n/a
Registration	27	In "closed" (non-open) surveys, users need to login first and it is easier to prevent duplicate entries from the same user. Describe how this was done. For example, was the survey never displayed a second time once the user had filled it in, or was the username stored together with the survey results and later eliminated? If the latter, which entries were kept for analysis (e.g., the first entry or the most recent)?	n/a
**ANALYSIS**			
Handling of incomplete responses	28	Were only completed questionnaires analyzed? Were questionnaires which terminated early (where, for example, users did not go through all questionnaire pages) also analyzed?	see 'Online survey' and 'Figure 7'
Questionnaires submitted with atypical timestamp	29	Some investigators may measure the time people needed to fill in a questionnaire and exclude questionnaires that were submitted too soon. Specify the timeframe that was used as a cut-off point and describe how this point was determined.	see 'Online survey' and 'Figure 7'
Statistical correction	30	Indicate whether any methods such as weighting of items or propensity scores have been used to adjust for the non-representative sample; if so, please describe the methods.	n/a

Supplementary Notes 1. Translated semi-structured interview guide for expert interviews with general practitioners.

Introduction of the interviewer and the research question

Introduction to the interview format and topics to be covered

Brief definition of the terms ‘digital maturity’ and ‘digital health solutions’

Part 1: Experience with digital health solutions ___________________________

- Could you tell me a little about yourself (your age, professional experience, place of work)?
- Could you tell me a little about your practice, including the type of practice and the patient population (statutory vs. privately health-insured patients)?
- Do you have any experience with digital health solutions (e.g., video consultations, electronic patient records, e-prescribing)?
- If so, which digital health solutions have you implemented in your practice?

Part 2: Perspectives on indicators of digital maturity

Part 3: Perceived barriers to the adoption of digital health solutions _________

- Are there aspects that currently hold you (or have held you) from implementing digital health solutions?
- If so, which aspects are these?
- In your opinion, what are the biggest barriers preventing general practitioners from using or adopting digital health solutions?

- On a scale of 1 (not relevant at all) to 10 (very relevant), how would you rate the relevance of barriers in the following categories concerning the adoption of digital health solutions in general practices?
    a. *Technological barriers*, i.e., barriers related to digital health solutions themselves or to technological aspects of the practice
    b. *Social barriers*, i.e., barriers related to general practitioners’ attitudes, skills, and interactions with patients
    c. *Organizational barriers*, i.e., barriers related to practice processes and workflows or the legal and political environment
- In the aforementioned categories or beyond – which other barriers do you think prevent general practitioners from using or adopting digital health solutions?

Part 4: Preferable strategies to improve digital health adoption

- How could the barriers you mentioned be overcome?
- What would encourage you (or has already encouraged you) to adopt digital health solutions?
- What could healthcare providers, policymakers, or other healthcare stakeholders do or what actions could they take to encourage you to adopt digital health solutions or support you in adoption?
- On a scale of 1 (no need for action at all) to 10 (paramount need for action), how would you rate the need for action towards the goal of implementing digital health solutions in the following areas?
    a. Development-related strategies, i.e., strategies related to improvements of the digital health solutions themselves, their development and adaptation
    b. Awareness-related strategies, i.e., strategies related to improving general practitioners’ awareness of digital health solutions
    c. Knowledge-related strategies, i.e., strategies related to increasing general practitioners’ knowledge of the functionalities, applicability, and underlying conditions of digital health solutions
    d. Implementation-related strategies, i.e., strategies related to improvements in the actual adoption of digital health solutions during implementation

e. Policy-related strategies, i.e., strategies related to improvements in regulatory requirements, guidelines, and legal regulation for digital health solutions

- In the aforementioned areas or beyond – which other activities or strategies do you think would support or encourage general practitioners to adopt digital health solutions?
- What kind of information would support your decision to adopt digital health solutions?

Conclusion and goodbye

Supplementary Notes 2. Translated survey questionnaire for general practitioners.

Disclaimer: The questionnaire was presented to respondents in German. This copy was translated, only stating questions included in the publication.

Introduction ____

Dear Sir or Madam,
Thank you for your interest in this study.

As part of a doctoral thesis at the Faculty of Health at Witten/Herdecke University, we are investigating the degree of digitalization of German general practices as well as your concerns and wishes regarding the adoption of digital health solutions. On this basis, we aim to derive relevant strategies that make it easier for general practitioners and decision-makers to advance the digitalization of the healthcare system.

In this study, we define digital health solutions as digital tools, technologies, and services to improve healthcare, make it more efficient, and personalize it. This includes the use of digital services (e.g., video consultations, digital telephone assistance system, digital appointment booking, digital medical history, digital practice administration) and the use of connected medical devices and artificial intelligence (e.g., telemonitoring, decision support systems).

This survey addresses practicing general practitioners in Germany. The survey will take about 10 to 15 minutes to complete. We would like to ask you to answer the questionnaire completely.

This research project has been approved by the Ethics Committee of Witten/Herdecke University (S-242/2022). On the following pages, you will find all the necessary information on data processing and your rights as a participant. To participate in this study, it is necessary that you electronically provide your informed consent afterward.

Thank you very much!

Information on data security and storage policies

Within this online survey, the personal data of the respondents will be collected and processed anonymously. The data collected is used purely for scientific purposes and cannot be traced to you personally. Due to the anonymous data collection, a subsequent deletion of your data is impossible. Your consent to the data protection regulations is voluntary and can be revoked in writing or verbally at any time without giving reasons and without personal disadvantage.

Show privacy policy.

☐ I have read the information on the survey data processing and agree to participate in the study.

Questions about yourself and your job

In the following, we would like to learn more about you as a person, your practice, and your interactions with digital health solutions.

By digital health solutions, we mean digital tools, technologies, and services to improve healthcare, make it more efficient, and personalize it. This includes the use of digital services (e.g., video consultations, digital telephone assistance system, digital appointment booking, digital medical history, digital practice administration) and the use of connected medical devices and artificial intelligence (e.g., telemonitoring, decision support systems).

Which gender do you identify with?	☐ male
	☐ female
	☐ diverse
	☐ no answer

How old are you?	☐ younger than 26 years old
	☐ 26 to 35 years old
	☐ 36 to 45 years old
	☐ 46 to 55 years old
	☐ 56 to 65 years old
	☐ older than 65 years old

Where do you work as a general practitioner?<br>I work in a village/town with…	☐ less than 5,000 inhabitants
	☐ 5,000 to 20,000 inhabitants
	☐ 20,001 to 100,000 inhabitants
	☐ 100,001 to 500,000 inhabitants
	☐ more than 500,000 inhabitants

How many years of professional experience do you currently have?	☐ less than 1 year
	☐ 1 to 5 years
	☐ 6 to 10 years
	☐ 11 to 20 years
	☐ 21 to 30 years
	☐ more than 30 years

Do you treat patients with statutory and/or private health insurance?	☐ only statutory health-insured patients
	☐ only privately health-insured patients
	☐ both

In which type of practice do you currently work?	☐ single practice
	☐ practice sharing
	☐ group practice
	☐ practice clinic
	☐ practice network
	☐ medical care center
	☐ collaborative laboratory

How often do you use digital health solutions?	☐ never
	☐ less than once per month
	☐ monthly
	☐ weekly
	☐ daily

How likely are you to use digital health solutions in the next 12 months?	☐ (1) very unlikely
	☐ (2) rather unlikely
	☐ (3) neither / nor
	☐ (4) rather likely
	☐ (5) very likely

How digitally affine do you consider the medical assistants in your practice to be in a professional context?	☐ (1) not at all digitally affine
	☐ (2) rather not digitally affine
	☐ (3) neither / nor digitally affine
	☐ (4) rather digitally affine
	☐ (5) fully digitally affine

Questions about your interaction with technical systems ___________________

In the following, we will ask you about your interaction with technical systems. The term 'technical systems' refers to apps and other software applications, as well as entire digital devices (e.g., mobile phone, computer, TV, car navigation).

Please indicate the degree to which you agree/disagree with the following statements.

	Completely disagree	Largely disagree	Slightly disagree	Slightly agree	Largely agree	Completely agree
I like to occupy myself in greater detail with technical systems.	☐	☐	☐	☐	☐	☐
I like testing the functions of new technical systems.	☐	☐	☐	☐	☐	☐
I predominantly deal with technical systems because I have to.	☐	☐	☐	☐	☐	☐
When I have a new technical system in front of me, I try it out intensively.	☐	☐	☐	☐	☐	☐
I enjoy spending time becoming acquainted with a new technical system.	☐	☐	☐	☐	☐	☐
It is enough for me that a technical system works; I don't care how or why.	☐	☐	☐	☐	☐	☐
I try to understand how a technical system exactly works.	☐	☐	☐	☐	☐	☐
It is enough for me to know the basic functions of a technical system.	☐	☐	☐	☐	☐	☐
I try to make full use of the capabilities of a technical system.	☐	☐	☐	☐	☐	☐

Questions about your personality

In the following, we would like to learn more about you as a person. For each of the following statements, please indicate how much it applies to you. You can grade your answers from "does not apply at all" to "fully applies".

	Does not apply at all	Does rather not apply	Neither / nor	Rather applies	Fully applies
I am rather shy, reserved.	☐	☐	☐	☐	☐
I tend to criticize others.	☐	☐	☐	☐	☐
I do tasks thoroughly.	☐	☐	☐	☐	☐
I become depressed easily.	☐	☐	☐	☐	☐
I have a wide range of interests.	☐	☐	☐	☐	☐
I am enthusiastic and can easily excite others.	☐	☐	☐	☐	☐
I trust others easily and believe in the good in people.	☐	☐	☐	☐	☐
I am comfortable and tend to be lazy.	☐	☐	☐	☐	☐
I am relaxed and don't let stress upset me.	☐	☐	☐	☐	☐
I am thoughtful and like to think about things.	☐	☐	☐	☐	☐
I am rather the 'quiet type' and taciturn.	☐	☐	☐	☐	☐
I can be cold and distant.	☐	☐	☐	☐	☐
I am efficient and work quickly.	☐	☐	☐	☐	☐
I worry a lot.	☐	☐	☐	☐	☐
I have an active imagination and am creative.	☐	☐	☐	☐	☐
I am outgoing and sociable.	☐	☐	☐	☐	☐
I can be brusque and dismissive towards others.	☐	☐	☐	☐	☐
I make plans and execute them.	☐	☐	☐	☐	☐
I become nervous and insecure easily.	☐	☐	☐	☐	☐
I appreciate artistic and aesthetic impressions.	☐	☐	☐	☐	☐
I have little artistic interest.	☐	☐	☐	☐	☐

Questions about the digital maturity of your practice (part 1) _______________

In the following, we would like to know more about your practice. For each of the following statements, please indicate how much you agree with them. You can grade your answers from “strongly disagree” to “strongly agree”.

	Strongly disagree	Rather disagree	Neither / nor	Rather agree	Strongly agree
In my practice, every team member is supported in the implementation of digital tools.	☐	☐	☐	☐	☐
My practice handles data and information responsibly and confidentially.	☐	☐	☐	☐	☐
I guide and lead team members as they implement new digital tools.	☐	☐	☐	☐	☐
In my practice, risks are actively identified and tracked to ensure information security.	☐	☐	☐	☐	☐
In my practice, standards and structures have been agreed upon and established.	☐	☐	☐	☐	☐
Our practice culture is participatory and inclusive and values innovation.	☐	☐	☐	☐	☐
My practice has the right hardware and network resources to use digital tools.	☐	☐	☐	☐	☐
The digital tools in my practice are of good quality.	☐	☐	☐	☐	☐
My practice offers video consultations.	☐	☐	☐	☐	☐
In my practice, patients can book, edit, and cancel appointments digitally.	☐	☐	☐	☐	☐
My practice uses an automated, digital telephone assistance system.	☐	☐	☐	☐	☐
My practice regularly uses e-prescriptions.	☐	☐	☐	☐	☐
My practice allows new patients to complete their medical history digitally.	☐	☐	☐	☐	☐
In my practice, billing and financial management are completely digital.	☐	☐	☐	☐	☐
My practice schedules, organizes, and records shifts and work hours digitally.	☐	☐	☐	☐	☐

Questions about the digital maturity of your practice (part 2) ________________

In the following, we would like to know more about your practice. For each of the following statements, please indicate how much you agree with them. You can grade your answers from “strongly disagree” to “strongly agree”.

	Strongly disagree	Rather disagree	Neither / nor	Rather agree	Strongly agree
My practice actively aims to provide ongoing training and education to team members.	☐	☐	☐	☐	☐
In my practice, team members actively share knowledge with each other.	☐	☐	☐	☐	☐
I use digital systems as intended.	☐	☐	☐	☐	☐
The team members in my practice use digital systems as intended.	☐	☐	☐	☐	☐
My practice digitally exchanges information with hospitals or other external partners.	☐	☐	☐	☐	☐
The digital systems within my practice are interconnected and exchange information.	☐	☐	☐	☐	☐
My practice operates flexibly and is able to adapt to internal or external changes.	☐	☐	☐	☐	☐
MY practice has a clear digital agenda that aligns with medical and business objectives.	☐	☐	☐	☐	☐
My practice analyzes and leverages existing patient data for effective decision making.	☐	☐	☐	☐	☐
My practice uses medical data to identify necessary adjustments in patient care early on.	☐	☐	☐	☐	☐
Our use of digital tools encourages patients to actively participate in health decisions.	☐	☐	☐	☐	☐
My practice involves patients in decisions about the implementation of digital tools.	☐	☐	☐	☐	☐
My practice shares data digitally with patients so they can view, record, and edit them.	☐	☐	☐	☐	☐

Questions about perceived barriers (part 1) ______________________________

In the following, we would like to learn more about barriers you perceive related to using or adopting digital health solutions.

As a reminder, we define digital health solutions (dhs) as digital tools, technologies, and services to improve healthcare, make it more efficient, and personalize it. This includes the use of digital services (e.g., video consultations, digital telephone assistance system, digital appointment booking, digital medical history, digital practice administration) and the use of connected medical devices and artificial intelligence (e.g., telemonitoring, decision support systems).

For each of the following statements, please indicate how much you agree with them. You can grade your answers from "strongly disagree" to "strongly agree".

	Strongly disagree	Rather disagree	Neither / nor	Rather agree	Strongly agree
I am skeptical about the design and technical capabilities of dhs.	☐	☐	☐	☐	☐
The use of dhs offers no additional value compared to my current practice processes.	☐	☐	☐	☐	☐
The use of dhs is relatively cumbersome and time-consuming.	☐	☐	☐	☐	☐
I am skeptical about the technical reliability of dhs.	☐	☐	☐	☐	☐
Dhs integrate rather poorly with existing practice software and tools.	☐	☐	☐	☐	☐
There is insufficient technical support from the provider when technical problems arise.	☐	☐	☐	☐	☐
The technical equipment in my practice is not sufficient for the implementation of dhs.	☐	☐	☐	☐	☐
I do not know which dhs are currently offered and what their goals and purposes are.	☐	☐	☐	☐	☐
I am not familiar with dhs and am unable to use them.	☐	☐	☐	☐	☐
I am skeptical and averse to the implementation of dhs.	☐	☐	☐	☐	☐
I am afraid that dhs will hinder my communication with patients.	☐	☐	☐	☐	☐
I am concerned that dhs will only be applicable or appropriate for some patients.	☐	☐	☐	☐	☐

Questions about perceived barriers (part 2) ______________________________

In the following, we would like to learn more about barriers you perceive related to using or adopting digital health solutions. For each of the following statements, please indicate how much you agree with them. You can grade your answers from "strongly disagree" to "strongly agree".

	Strongly disagree	Rather disagree	Neither / nor	Rather agree	Strongly agree
I do not believe that dhs will improve my patients' access to care.	☐	☐	☐	☐	☐
My patients are not interested in or able to use dhs.	☐	☐	☐	☐	☐
I am concerned that dhs may have negative medical consequences.	☐	☐	☐	☐	☐
The adoption of dhs requires adjustments to existing practice processes and workflows.	☐	☐	☐	☐	☐
The implementation of dhs is costly and subsequent use is not adequately reimbursed.	☐	☐	☐	☐	☐
There is insufficient empirical evidence about the benefits of dhs for patients.	☐	☐	☐	☐	☐
I do not have sufficient information on existing dhs and how they work.	☐	☐	☐	☐	☐
The implementation of dhs will lead to an increased workload.	☐	☐	☐	☐	☐
The implementation of dhs will require a high training and familiarization effort.	☐	☐	☐	☐	☐
I do not have a strategic plan for the introduction of dhs.	☐	☐	☐	☐	☐
There are no clear and adequate regulations, guidelines, and health policies around dhs.	☐	☐	☐	☐	☐
I am skeptical about the confidentiality and security of personal information related to dhs.	☐	☐	☐	☐	☐
I am concerned about medicolegal risks related to dhs, e.g., liability risks.	☐	☐	☐	☐	☐
I have insufficient time to implement and use dhs.	☐	☐	☐	☐	☐

Questions about potential measures to support adoption (part 1) ____________

In the following, we would like to learn more about which aspects might encourage you to implement digital health solutions in your practice or might support you in doing so.

As a reminder, we define digital health solutions (dhs) as digital tools, technologies, and services to improve healthcare, make it more efficient, and personalize it. This includes the use of digital services (e.g., video consultations, digital telephone assistance system, digital appointment booking, digital medical history, digital practice administration) and the use of connected medical devices and artificial intelligence (e.g., telemonitoring, decision support systems).

For each of the following statements, please indicate how much you agree with them. You can grade your answers from “strongly disagree” to “strongly agree”.

I would be more likely to adopt or use digital health solutions, …	Strongly disagree	Rather disagree	Neither / nor	Rather agree	Strongly agree
…if I were involved in the design, planning and realization of dhs.	☐	☐	☐	☐	☐
…if providers improved the benefits for my practice and my patients.	☐	☐	☐	☐	☐
…if providers optimized user-friendliness and ease of use.	☐	☐	☐	☐	☐
…if providers improved integrability with existing software and tools.	☐	☐	☐	☐	☐
…if user feedback were incorporated into the development and improvement of dhs.	☐	☐	☐	☐	☐
…if there was the possibility to customize and individualize dhs for my practice.	☐	☐	☐	☐	☐
…if I had an overview of dhs offered, their goals, and their intended use.	☐	☐	☐	☐	☐
…if there were recommendations from medical associations or scientific societies.	☐	☐	☐	☐	☐
…if colleagues or important opinion leaders would report about positive experiences.	☐	☐	☐	☐	☐
…if patients would like to see the use or introduction of dhs.	☐	☐	☐	☐	☐

Questions about potential measures to support adoption (part 1) ___________

In the following, we would like to learn more about which aspects might encourage you to implement digital health solutions in your practice or might support you in doing so. For each of the following statements, please indicate how much you agree with them. You can grade your answers from “strongly disagree” to “strongly agree”.

I would be more likely to adopt or use digital health solutions, …	Strongly disagree	Rather disagree	Neither / nor	Rather agree	Strongly agree
…if I had more information about the functionalities and integration into existing workflows.	☐	☐	☐	☐	☐
…if I had more information about the potential benefits for physicians and for patients.	☐	☐	☐	☐	☐
…if there was additional research and information about the benefits and potential risks.	☐	☐	☐	☐	☐
…if I had more information about available reimbursement and financing models.	☐	☐	☐	☐	☐
…if I had the opportunity to test dhs before implementation as part of a trial version.	☐	☐	☐	☐	☐
…if I received continuous technical support from the provider before and after rollout.	☐	☐	☐	☐	☐
…if the provider would offer reliable training material on the use of the dhs.	☐	☐	☐	☐	☐
…if I received regular training on dhs as part of my continuing professional education.	☐	☐	☐	☐	☐
…if there were educational opportunities for patients about dhs and associated benefits.	☐	☐	☐	☐	☐
…if competencies in the use of dhs were included in medical professional training.	☐	☐	☐	☐	☐
…if there were financial incentives for the introduction or the reimbursement were improved.	☐	☐	☐	☐	☐
…if policies and regulations were simplified and there were guidelines on medico-legal risks.	☐	☐	☐	☐	☐
…if there were legal regulations that simplified data protection and handling of personal data.	☐	☐	☐	☐	☐

Conclusion and goodbye

You have reached the end of this survey. Your responses have now been recorded.

Thank you very much for your participation!

Do you have colleagues who might also be interested in participating? If so, please forward the survey link to them: survey link.

You may now close the browser window.
